# Cardiovascular magnetic resonance in the guidelines of the European Society of Cardiology: a comprehensive summary and update

**DOI:** 10.1186/s12968-023-00950-z

**Published:** 2023-07-24

**Authors:** Florian von Knobelsdorff-Brenkenhoff, Jeanette Schulz-Menger

**Affiliations:** 1 KIZ-Kardiologie Im Zentrum, and Ludwig-Maximilians-University, Munich, Germany; 2 Working Group Cardiovascular Magnetic Resonance, Experimental and Clinical Research Center, a Joint Cooperation Between the Charité Medical Faculty and the Max-Delbrueck Center for Molecular Medicine, Berlin, Germany; 3 Department of Cardiology and Nephrology, HELIOS Klinikum Berlin Buch, Lindenberger Weg 80, 13125 Berlin, Germany

**Keywords:** Cardiovascular magnetic resonance, Guideline, ESC

## Abstract

**Background:**

Cardiovascular magnetic resonance (CMR) has been established as a valuable tool in clinical and scientific cardiology. This study summarizes the current evidence and role of CMR in the guidelines of the European Society of Cardiology (ESC) and is an update of a former guideline analysis.

**Methods:**

Since the last guideline analysis performed in 2015, 28 new ESC guideline documents have been published. Twenty-seven ESC practice guidelines are currently in use. They were screened regarding CMR in the text, tables and figures. The main CMR-related sentences and recommendations were extracted.

**Results:**

Nineteen of the 27 guidelines (70.4%) contain relevant text passages regarding CMR in the text and include 92 specific recommendations regarding the use of CMR. Seven guidelines (25.9%) mention CMR in the text, and 1 (3.7%, dyslipidemia) does not mention CMR. The 19 guidelines with recommendations regarding the use of CMR contain 40 class-I recommendations (43.5%), 28 class-IIa recommendations (30.4%), 19 class-IIb recommendations (20.7%) and 5 class-III recommendations (5.4%). Most of the recommendations have evidence level C (56/92; 60.9%), followed by level B (34/92; 37.0%) and level A (2/92; 2.2%). Twenty-one recommendations refer to the field of cardiomyopathies, 21 recommendations to stress perfusion imaging, 20 recommendations to vascular assessment, 12 to myocardial tissue characterization in general, 8 to left and right ventricular function assessment, 5 to the pericardium and 5 to myocarditis.

**Conclusions:**

CMR is integral part of the majority of the ESC guidelines. Its representation in the guidelines has increased since the last analysis from 2015, now comprising 92 instead of formerly 63 specific recommendations. To enable patient management in accordance to the ESC guidelines, CMR must become more widely available.

**Supplementary Information:**

The online version contains supplementary material available at 10.1186/s12968-023-00950-z.

## Background

Cardiovascular magnetic resonance (CMR) is established in a wide variety of indications in clinical cardiology, most frequently inflammatory and ischemic heart disease as well as cardiomyopathies. CMR provides detailed information about cardiovascular anatomy and function by combining diverse techniques, including determination of cardiac size and contraction, oedema, fibrosis, and perfusion.

Despite common sense about the diagnostic value of CMR, the access to CMR is quite diverse, both differing between the various countries, but even regionally within nations. This limitation is often attributed to missing access to scanners with cardiac equipment, missing skills to run and interpret CMR, as well as differing health insurance systems dealing differently with the procedural costs and reimbursement.

The guidelines of the European Society of Cardiology are highly important, globally applied documents that were produced after careful consideration of the scientific and medical knowledge and the evidence available at the time of their publication. In 2016, we published a systematic analysis of the representation of CMR in these guidelines, demonstrating that most guidelines already contained recommendations to perform CMR in various clinical scenarios [1]. This former analysis contributed to the discussion about the use, training, distribution and reimbursement of CMR in Europe. Since then, 28 new and updated guideline documents have been published by the ESC. This analysis is a comprehensive summary and update about the role of CMR in the ESC guidelines and aims to guide evidence-based application of CMR.

## Methods

All ESC guidelines listed on the ESC website (https://www.escardio.org/Guidelines) were collected (Table 1). If more than one guideline for the same topic had been published in the past, the most recent was included in the final analysis. The documents were screened for the terms “magnetic”, “MRI”, “CMR”, “MR”, “imaging”, “stress”, “LGE”, “fibrosis”, “perfusion”, and if present, the relation to CMR was evaluated. The main paragraphs with referral to CMR were extracted. If the guideline contained specific recommendations with referral to CMR, the recommendation was summarized in tables with level of evidence and class of recommendation as established in the ESC guidelines (Table 2). If a recommendation referred to “imaging” in general, it was registered if the context included CMR. MRI in the context of non-cardiovascular examinations like brain MRI was not included. Specific recommendations and texts were predominantly taken over literally from the guideline into this paper to keep with the comprehensive wordings worked out by the corresponding guideline task forces. The results were compared to the former guideline versions. Guidelines other than by the ESC as well as ESC position statements were not considered. ESC Clinical Practice Guidelines are developed following ESC policies and procedures that are available online (“guideline development”). The composition and selection of the task force members that develop the practice guideline follows predefined rules, considers authors with specific expertise in the guideline topic and integrates the ESC subspecialty communities. The European Association of Cardiovascular Imaging (EACVI) has participated in the development of all but one (“syncope”) ESC practice guideline document that are included in this analysis.

**Table 1:** List of ESC guidelines used for the analysis

Nr	Guideline keyword	Year of publication	Representation of CMR in the guideline	Class of recommendation			
				I	IIa	IIb	III
1	Ventricular arrhythmias and sudden cardiac death [2]	2022	CMR mentioned in the guideline text and in 17 recommendations	4	10	3	0
2	Non-cardiac surgery: cardiovascular assessment [3]	2022	CMR mentioned in the guideline text and in 4 recommendations	1	1	1	1
3	Cardio-oncology [4]	2022	CMR mentioned in the guideline text and in 5 recommendations	4	1	0	0
4	Pulmonary hypertension [5]	2022	CMR mentioned in the guideline text and in 1 recommendation	0	0	1	0
5	Valvular heart disease [6]	2021	CMR mentioned in the guideline text	-	-	-	-
6	Prevention [7]	2021	CMR mentioned in the guideline text	-	-	-	-
7	Pacing [8]	2021	CMR mentioned in the guideline text and in 2 recommendations	1	1	0	0
8	Heart failure [9]	2021	CMR mentioned in the guideline text and in 4 recommendations	2	1	1	0
9	Sports [10]	2020	CMR mentioned in the guideline text and in 5 recommendations	1	2	1	1
10	NSTEMI [11]	2020	CMR mentioned in the guideline text and in 1 recommendation	1	0	0	0
11	Atrial fibrillation [12]	2020	CMR mentioned in the guideline text	–	–	–	–
12	Congenital heart disease [13]	2020	CMR mentioned in the guideline text and in 3 recommendations	1	2	0	0
13	Chronic coronary syndrome [14]	2019	CMR mentioned in the guideline text and in 10 recommendations	4	1	3	2
14	Diabetes [15]	2019	CMR mentioned in the guideline text and in 3 recommendations	1	0	2	0
15	Dyslipidaemias [16]	2019	CMR not mentioned in the guideline text	–	–	–	–
16	Pulmonary embolism [17]	2019	CMR mentioned in the guideline text and in 1 recommendation	0	0	0	1
17	Supraventricular tachycardia [18]	2019	CMR mentioned in the guideline text	–	–	–	–
18	Myocardial revascularization [19]	2018	CMR mentioned in the guideline text and in 4 recommendation	0	1	3	0
19	Arterial hypertension [20]	2018	CMR mentioned	–	–	–	–
20	Cardiovascular diseases in pregnancy [21]	2018	CMR mentioned in the guideline text and in 3 recommendations	2	1	0	0
21	Syncope [22]	2018	CMR mentioned in the guideline text	–	–	–	–
22	Peripheral artery disease [23]	2017	CMR mentioned in the guideline text and in 5 recommendations	5	0	0	0
23	STEMI [24]	2017	CMR mentioned in the guideline text and in 3 recommendations	0	2	1	0
24	Pericardial diseases [25]	2015	CMR mentioned in the guideline text and in 5 recommendations	3	1	1	0
25	Endocarditis [26]	2015	CMR mentioned in the guideline text	–	–	–	–
26	Hypertrophic cardiomyopathy [27]	2014	CMR mentioned in the guideline text and in 7 recommendations	2	3	2	0
27	Aortic diseases [28]	2014	CMR mentioned in the guideline text and in 9 recommendations	8	1	0	0

*ESC* European Society of Cardiology, *CMR* Cardiovascular magnetic resonance, *NSTEMI* Non ST elevation myocardial infarction, *STEMI* ST elevation myocardial infarction

**Table 2:** Class of recommendation and level of evidence as defined in the ESC guidelines

Class of recommendation	Definition
I	Evidence and/or general agreement that a given treatment or procedure is beneficial, useful, effective
II	Conflicting evidence and/or a divergence of opinion about the usefulness/efficacy of the given treatment or procedure
IIa	Weight of evidence/opinion is in favor of usefulness/efficacy
IIb	Usefulness/efficacy is less well established by evidence/opinion
III	Evidence or general agreement that the given treatment or procedure is not useful/effective, and in some cases may be harmful

Level of evidence	Definition
A	Data derived from multiple randomized clinical trials or meta-analyses
B	Data derived from a single randomized clinical trial or large non-randomized studies
C	Consensus of opinion of the experts and/ or small studies, retrospective studies, registries

*ESC* European Society of Cardiology

## Results

### Results across all guidelines

Of the 27 ESC guidelines, 19 (70.4%) guidelines contain relevant text passages with referral to CMR and 92 specific recommendations regarding the use of CMR in various scenarios (Table 1). Seven guidelines (25.9%) mention CMR in the text but do not contain specific recommendations that contain CMR. One guideline (3.7%; dyslipidaemia) does not mention CMR throughout the guideline.

**Fig. 1:**
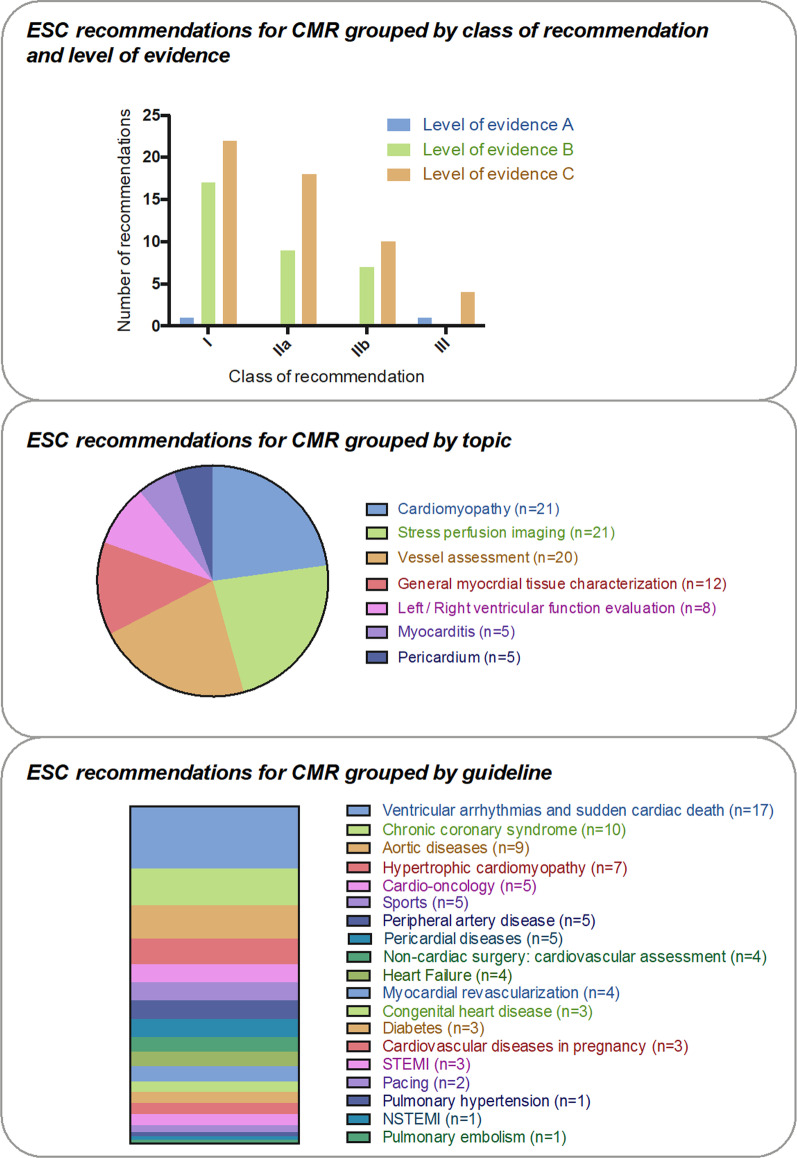
Summary of the specific ESC recommendations with referral to CMR

The 19 guidelines with specific recommendations regarding the use of CMR contain 40 class-I recommendations (43.5%), 28 class-IIa recommendations (30.4%), 19 class-IIb recommendations (20.7%) and 5 class-III recommendations (5.4%). Most of the recommendations have evidence level C (56/92; 60.9%), followed by level B (34/92; 37.0%) and level A (2/92; 2.2%) (Fig. 1).

Twenty-one recommendations refer to the field of cardiomyopathies, 21 recommendations to stress perfusion imaging, 20 recommendations to vessel assessment, 12 to myocardial tissue characterization in general, 8 to left and right ventricular function assessment, 5 to the pericardium and 5 to myocarditis. The five guidelines which contained the highest number of recommendations for CMR, were ventricular arrhythmias and sudden cardiac death (n = 17), chronic coronary syndrome (n = 10), aortic diseases (n = 9), HCM (n = 7) and cardio-oncology (n = 5) (Fig. 1).

### Results per individual guideline

#### 2022 ESC Guidelines for the management of patients with ventricular arrhythmias and the prevention of sudden cardiac death [2]

##### CMR in specific recommendations

This guideline contains 17 recommendations with referral to CMR: 4 × class I, 10 × class IIa, 3 × class IIb (Table 3).

**Table 3:** Recommendations for CMR in the guidelines for the management of patients with ventricular arrhythmias and the prevention of sudden cardiac death

Recommendation	Class	Level
*Recommendations for evaluation of patients presenting with newly documented ventricular arrhythmia*		
In patients with newly documented ventricular arrhythmia (frequent premature ventricular contractions (PVCs), non-sustained ventricular tachycardia (NSVT), sustained monomorphic ventricular tachycardia (SMVT) and suspicion of structural heart disease other than coronary artery disease after initial evaluation, a CMR should be considered	IIa	B
*Recommendations for evaluation of sudden cardiac arrest survivors*		
Coronary imaging and CMR with LGE are recommended for evaluation of cardiac structure and function in all sudden cardiac arrest survivors without a clear underlying cause	I	B
*Recommendations for evaluation of relatives of sudden arrhythmic death syndrome decedents*		
Ambulatory cardiac rhythm monitoring and CMR may be considered in relatives of sudden arrhythmic death syndrome (SADs) decedents	IIb	C
*Recommendations for the management of patients with idiopathic premature ventricular complexes/ventricular tachycardia*		
In patients with PVCs / ventricular tachycardia (VT) and a presentation not typical for an idiopathic origin, CMR should be considered, despite a normal echocardiogram	IIa	C
*Recommendations for the management of patients with premature ventricular complex-induced or premature ventricular complex aggravated cardiomyopathy*		
In patients with suspected PVCs- induced cardiomyopathy, CMR should be considered	IIa	B
*Recommendations for risk stratification, sudden cardiac death prevention, and treatment of ventricular arrhythmias in dilated cardiomyopathy (DCM) / hypokinetic non-dilated cardiomyopathy (HNDCM)*		
CMR with LGE should be considered in dilated cardiomyopathy (DCM) / hypokinetic non-dilated cardiomyopathy (HNDCM) patients for assessing the aetiology and the risk of ventricular arrhythmia (VA) / sudden cardiac death (SCD)	IIa	B
ICD implantation should be considered in DCM / HNDCM patients with a LVEF < 50% and ≥ 2 risk factors (syncope, LGE on CMR, inducible sustained monomorphic VT (SMVT) at programmed electrical stimulation (PES), pathogenic mutations in *LMNA*, *PLN*, *FLNC*, and *RBM 20* genes)	IIa	C
*Recommendations for diagnostic, risk stratification, sudden cardiac death prevention, and treatment of ventricular arrhythmias in arrhythmogenic right ventricular cardiomyopathy*		
In patients with suspected arrhythmogenic right ventricular cardiomyopathy (ARVC), CMR is recommended	I	B
*Recommendations for risk stratification, sudden cardiac death prevention, and treatment of ventricular arrhythmias in hypertrophic cardiomyopathy*		
CMR with LGE is recommended in hypertrophic cardiomyopathy (HCM) patients for diagnostic work-up	I	B
ICD implantation should be considered in HCM patients aged 16 years or more with an intermediate 5-year risk of SCD (≥ 4 to < 6%) and with (a) significant LGE at CMR (usually ≥ 15% of LV mass); or (b) LVEF < 50%; or (c) abnormal blood pressure response during exercise test; or (d) LV apical aneurysm; or (e) presence of sarcomeric pathogenic mutation	IIa	B
ICD implantation may be considered in HCM patients aged 16 years or more with a low estimated 5-year risk of SCD (< 4%) and with (a) significant LGE at CMR (usually ≥ 15% of LV mass); or (b) LVEF < 50%; or (c) LV apical aneurysm	IIb	B
*Recommendations for implantable cardioverter defibrillator implantation in left ventricular non-compaction*		
In patients with a left ventricular non-compaction (LVNC) cardiomyopathy phenotype based on CMR or echocardiography, implantation of an ICD for primary prevention of SCD should be considered to follow DCM / HNDCM recommendations	IIa	C
*Recommendations for risk stratification, sudden cardiac death prevention, and treatment of ventricular arrhythmias in neuromuscular diseases*		
Invasive electrophysiological evaluation should be considered in patients with myotonic dystrophy and a PR interval ≥ 240 ms or QRS duration ≥ 120 ms or who are older than 40 years and have supraventricular arrhythmias or who are older than 40 years and have significant LGE on CMR	IIa	B
Implantation of an ICD may be considered in patients with Duchenne/Becker muscular dystrophy and significant LGE at CMR	IIb	C
*Recommendations for risk stratification, sudden cardiac death prevention, and treatment of ventricular arrhythmias in cardiac sarcoidosis*		
In patients with cardiac sarcoidosis who have a LVEF > 35% but significant LGE at CMR after resolution of acute inflammation, ICD implantation should be considered	IIa	B
In patients with cardiac sarcoidosis who have a LVEF 35–50% and minor LGE at CMR, after resolution of acute inflammation, programmed electrical stimulation (PES) for risk stratification should be considered	IIa	C
*Recommendations for risk stratification and prevention of sudden cardiac death in athletes*		
In athletes with positive medical history, abnormal physical examination, or ECG alterations, further investigations including echocardiography and/or CMR to confirm (or exclude) an underlying disease are recommended	I	C

*CMR* cardiovascular magnetic resonance, *LGE* Late Gadolinium Enhancement, *LVEF* left ventricular ejection fraction, *ICD* implantable cardioverter defibrillator, *ECG* electrocardiogram

##### CMR in the guideline text

Initially, the strength of CMR is highlighted generally: “CMR currently provides the most accurate and reproducible measurement of atrial, biventricular global and regional systolic function, and can detect myocardial oedema, fibrosis, infiltration, and perfusion defects. CMR is more sensitive than echocardiography to diagnose arrhythmogenic right ventricular cardiomyopathy (ARVC), is diagnostic in left ventricular non-compaction (LVNC), and can detect apical aneurysms in hypertrophic cardiomyopathy (HCM). Fibrosis detection by LGE contributes to ventricular arrhythmia risk stratification in HCM, dilated cardiomyopathy (DCM) and potentially in mitral valve prolapse arrhythmic syndrome. Novel myocardial mapping techniques can detect diffuse fibrosis and can suggest the etiology of left ventricular hypertrophy for specific therapy guiding, e.g. Fabry disease and amyloidosis. The prognostic value remains to be evaluated.”

Then, CMR is specified for various arrhythmic scenarios. Upon first presentation with ventricular arrhythmia in patients without known cardiac disease “CMR should be considered when cardiomyopathies or inflammatory diseases are suspected on initial evaluation. In addition, CMR can identify areas of fibrosis as substrates of non-sustained ventricular tachycardia (NSVT).” In case of first presentation of sustained monomorphic ventricular tachycardia (SMVT), “atypical ECG morphologies and uncommon clinical presentations should raise suspicions for underlying structural heart disease. In this scenario, additional evaluation with CMR should be considered. < … > If ECG and echocardiography are suggestive for a cardiomyopathy, CMR provides important diagnostic information on scar distribution and tissue characteristics.” In sudden cardiac arrest survivors, “CMR has repeatedly been shown to provide significant incremental diagnostic value, in particular for concealed cardiomyopathy.” In relatives of sudden arrhythmic death syndrome decedents, CMR may be helpful if the results of the baseline cardiac tests diverge. In idiopathic premature ventricular complexes (PVC) / ventricular tachycardia (VT), “CMR should be performed whenever ECG and echocardiography are inconclusive to rule out structural heart disease, or the clinical presentation raises suspicion for structural heart disease.” CMR should be considered for patients suspected to have premature ventricular complex-induced cardiomyopathy to exclude subtle forms of structural heart disease (SHD). “In a patient with frequent PVCs, the presence of LGE suggests SHD with frequent PVCs rather than PVC-induced cardiomyopathy, in which LGE is mostly absent. Given that PVCs with a right bundle branch block (RBBB) morphology have been reported to show a stronger association with LGE, those patients should be particularly considered for CMR. “When planning VT ablation, it is important to collect all available information about the arrhythmogenic substrate, especially to identify scars (using CMR).” In PVCs, factors affecting acute ablation success and clinical outcome include < … > the absence of LGE on CMR.”

For risk stratification and primary prevention of sudden cardiac death in patients with DCM / hypokinetic non-dilated cardiomyopathy (HNDCM), careful diagnostic work-up, including genetic testing and CMR, should be considered to identify the underlying cause for etiology-oriented risk stratification and treatment. “Discrimination between high- and low-risk patients for SCD remains challenging. Beyond LVEF and NYHA class, recent data suggest that both genetic and CMR findings can contribute to risk stratification.” In ARVC, tissue characterization by CMR was not included in the 2010 revised international diagnostic task force criteria. “However, right ventricular fatty infiltration and left ventricular LGE are frequently observed < … > and may be present before patients meet major task force imaging criteria.” Therefore, “In patients with suspected ARVC, CMR is recommended.” In suspected HCM, as the natural history and management differs according to the underlying etiology of left ventricular hypertrophy, “diagnostic work-up is of paramount importance and includes CMR and genetic testing”. Regarding risk stratification and primary prevention of sudden cardiac death in HCM, “additional factors not captured by the Risk-SCD model should also be considered in patients with intermediate or low calculated risk, including < … > LGE on CMR < … > . Extensive LGE on CMR defined as ≥ 15% of LV mass has been suggested as good predictor of sudden cardiac death in adults.” In left ventricular non-compaction, “CMR-based detection of focal fibrosis using LGE was associated with serious cardiac events. < … > A combination of CMR criteria with systematic genotyping may overcome current uncertainties regarding risk stratification.” In neuromuscular disorders, LGE at CMR is associated with increased risk of atrioventricular block and SCD and may influence the decision for implantation of a pacemaker or ICD. In myocarditis, CMR adds to make the clinical diagnosis. “In patients with SMVT of unclear etiology, myocarditis should be suspected especially when CMR reveals subepicardial and/or intramural abnormal fibrotic myocardial tissue. The presence of LGE at CMR has also been associated with the late occurrence of ventricular arrhythmias in patients with endomyocardial biopsy-proven viral myocarditis.” Subclinical cardiac sarcoidosis is increasingly diagnosed using advanced cardiac imaging modalities (CMR/PET-CT). In cardiac sarcoidosis, presence of right or left ventricular scarring at CMR has been found associated with an adverse outcome and helps stratify the risk of ventricular arrhythmia. Whatever the LVEF, ICD should be considered in patients with an indication for permanent cardiac pacing or the presence of significant scar at CMR. In Chagas’ disease, “the presence of myocardial fibrosis at LGE-CMR is useful in assessing the risk of death.” In mitral valve prolapse, “myocardial fibrosis affecting both the infero-basal LV free wall and the papillary muscles has been recognized in pathological and LGE-CMR studies. < … > This indicates a promising role of CMR for arrhythmic risk stratification. Myocardial fibrosis on CMR may add to the risk profile” of ‘arrhythmic mitral valve prolapse syndrome’. In congenital heart disease, patients with sustained ventricular arrhythmia or cardiac arrest survivors, a comprehensive evaluation of inciting factors, including cardiac imaging (particularly CMR) < … > is important. The diagnosis of idiopathic ventricular fibrillation is made in cardiac arrest survivors, preferably with documented ventricular fibrillation, after exclusion of structural, channelopathic, metabolic, or toxicological aetiologies. Diagnostic tests include < among others > CMR.” For risk stratification and prevention of sudden cardiac death in athletes, “additional tests, such as echocardiography, 24-h Holter monitoring, stress testing, and CMR, are requested for athletes who had positive findings at the initial evaluation.”

##### Comparison between the current and the last guideline

The guideline from 2015 already mentioned CMR in the text as a valuable tool in many scenarios. The current guideline from 2022 specifies these scenarios and more frequently defines recommendations. The number of specific recommendations has increased from 4 in 2015 to 17 in 2022.

#### 2022 ESC Guidelines on cardiovascular assessment and management of patients undergoing non-cardiac surgery [3]

##### CMR in specific recommendations

This guideline contains 4 recommendations with referral to CMR: 1 × class I, 1 × class IIa, 1 × class IIb and 1 × class III (Table 4).

**Table 4:** Recommendations for CMR in the guidelines on cardiovascular assessment and management of patients undergoing non-cardiac surgery

Recommendation	Class	Level
*Recommendations for stress imaging*		
Stress imaging is recommended before high-risk elective non-cardiac surgery in patients with poor functional capacity and high likelihood of coronary artery disease or high clinical risk	I	B
Stress imaging should be considered before high-risk non-cardiac surgery in asymptomatic patients with poor functional capacity, and previous percutaneous coronary intervention (PCI) or coronary artery bypass graft (CABG)	IIa	C
Stress imaging may be considered before intermediate-risk non-cardiac surgery when ischemia is of concern in patients with clinical risk factors and poor functional capacity	IIb	B
Stress imaging is not recommended routinely before non-cardiac surgery	III	C

*CMR*  cardiovascular magnetic resonance

##### CMR in the guideline text

To assess perioperative risk, stress imaging is recommended in various scenarios. Different stress imaging methods are named including CMR. “Stress CMR imaging and late gadolinium enhancement are also accurate tools for detection of ischemic heart disease and prognostication.”

##### Comparison between the current and the last guideline

The current guideline contains 4 and the former guideline from 2014 contained 3 specific recommendations with referral to CMR as stress imaging method. In general, the role of CMR remained constant between both guidelines.

#### 2022 ESC Guidelines on cardio-oncology [4]

##### CMR in specific recommendations

This guideline contains 5 recommendations (4 × class I, 1 × class IIa) with referral to CMR (Table 5):

**Table 5:** Recommendations for CMR in the guidelines on cardio-oncology

Recommendation	Class	Level
*Recommendations for cardiac imaging modalities in patients with cancer*		
CMR should be considered for the assessment of cardiac function when echocardiography is unavailable or non-diagnostic	IIa	C
*Recommendations for the diagnosis and management of immune checkpoint inhibitor-associated myocarditis*		
Cardiac Troponin (cTn), ECG, and cardiovascular imaging (echocardiography and CMR) are recommended to diagnose immune checkpoint inhibitor (ICI)—associated myocarditis	I	B
*Recommendations for the diagnosis and management of Takotsubo syndrome in patients with cancer*		
CMR is recommended to exclude myocarditis and myocardial infarction	I	B
*Recommendations for the management of pericardial diseases in patients receiving anticancer treatment*		
Multimodality cardiovascular imaging (echocardiography, CMR ± CT), ECG and measurement of cardiac biomarkers are recommended to confirm the diagnosis, assess the hemodynamic consequences of pericardial disease, and rule out associated myocarditis	I	C
*Recommendations for amyloid light-chain cardiac amyloidosis diagnosis and monitoring*		
CMR is recommended in patients with suspected amyloid light-chain cardiac amyloidosis (AL-CA)	I	A

*CMR*  cardiovascular magnetic resonance, *ECG*  electrocardiogram, *CT*  computed tomography

##### CMR in the guideline text

“Cardiovascular imaging has an important role in identifying patients with subclinical cardiovascular disease, determining the degree of pre-existing cardiac comorbidity prior to decisions regarding cancer therapy, and serves as a reference for identification of changes during treatment and long-term follow-up.” “Imaging techniques—particularly advanced echocardiography and CMR—facilitate early diagnosis and management of cancer-therapy-related cardiovascular toxicity”.

CMR is mentioned in many scenarios: In subjects with poor-quality echocardiography windows, when available, CMR should be considered to assess cardiac volumes and function. CMR may be helpful for identifying intracardiac masses on readily available routine imaging performed for cancer staging. If a specific cardiovascular disease is identified (e.g. hypertrophic cardiomyopathy), CMR should be considered for further risk assessment. Functional imaging tests for myocardial ischemia including perfusion CMR should be performed to assess for ischemia in symptomatic patients if clinical suspicion of coronary artery disease exists. In patients with immune checkpoint inhibitors, CMR is recommended when myocarditis is suspected. Investigations in a patient with cancer with suspected Takotsubo cardiomyopathy should include CMR to rule out myocarditis and myocardial infarction. In case of myocardial infarction with non-obstructive coronary arteries, CMR may be considered to detect other causes of myocardial injury, especially myocarditis and Takotsubo cardiomyopathy. Patients with systemic embolization may be screened with CMR, “as it is more sensitive and specific than TTE for detecting intracardiac thrombi and late gadolinium enhancement CMR is currently considered the gold standard”. In suspected pericardial disease, “CMR can provide additional information on pericardial inflammation and constrictive physiology”. In cardiac tumors, CMR is recommended “for cardiac tumor tissue characterization”. In suspected amyloid light-chain amyloidosis, “CMR with LGE and parametric imaging has emerged as a new non-invasive gold-standard.”

##### Comparison between the current and the last guideline

The cardio-oncology guideline is a completely new guideline. A comparison with previous versions is not possible.

#### 2022ESC/ERS Guidelines for the diagnosis and treatment of pulmonary hypertension [5]

##### CMR in specific recommendations

This guideline contains 1 recommendation (class IIb) with referral to CMR (Table 6).

**Table 6:** Recommendations for CMR in the guidelines for the diagnosis and treatment of pulmonary hypertension

Recommendation	Class	Level
*Recommendations for screening and improved detection of pulmonary arterial hypertension and chronic thrombo-embolic pulmonary hypertension*		
In symptomatic patients with systemic sclerosis, exercise echocardiography or cardiopulmonary exercise testing (CPET), or CMR may be considered to aid decisions to perform right heart catheterization	IIb	C

*CMR*  cardiovascular magnetic resonance

##### CMR in the guideline text

CMR “accurately and reproducibly assesses atrial and ventricular size, morphology, and function. Additional information on right / left ventricular (RV / LV) myocardial strain can be obtained by applying tagging or by post-processing feature tracking. In addition, CMR can be used to measure blood flow in the pulmonary artery, aorta, and vena cava, allowing for quantifying stroke volume, intracardiac shunt, and retrograde flow. By combining contrast magnetic resonance angiography and pulmonary perfusion imaging with late gadolinium-enhancement imaging of the myocardium, a complete picture of the heart and pulmonary vasculature can be obtained. < … > Even though the cost and availability of the technique precludes its use in the early diagnosis of pulmonary artery hypertension (PAH), it is sensitive in detecting early signs of pulmonary hypertension and diagnosing congenital heart disease.” “The role of CMR in evaluating patients with PAH has been addressed in several studies, and RV volumes, RV-EF, and RV stroke volume are essential prognostic determinants in PAH. CMR enables treatment effects to be monitored and treatment strategies adapted in time to prevent clinical failure.**”**

##### Comparison between the current and the last guideline

Compared to the guideline from 2015, the guideline from 2022 for the first time contains a specific recommendation for CMR in systemic sclerosis. Furthermore, the risk-stratification table has been expanded including additional CMR prognostic indicators.

#### 2021 ESC/EACTS Guidelines for the management of valvular heart disease [6]

##### CMR in the guideline text

“Non-invasive evaluation using three-dimensional echocardiography, cardiac computed tomography (CCT), CMR, and biomarkers plays a more and more central role.” “Multimodality imaging including echocardiography, CCT, CMR, and nuclear medicine”, is listed as necessary requirement for a Heart Valve Centre. In general, “in patients with inadequate echocardiographic quality or discrepant results, CMR should be used to assess the severity of valvular lesions, particularly regurgitant lesions, and to assess ventricular volumes, systolic function, abnormalities of the ascending aorta, and myocardial fibrosis. CMR is the reference method for the evaluation of right ventricular volumes and function and is therefore particularly useful to evaluate the consequences of tricuspid regurgitation. It also has an incremental value for assessing the severity of aortic and mitral regurgitation.”

In aortic regurgitation, “CMR should be used to quantify the regurgitant fraction when echocardiographic measurements are equivocal or discordant with clinical findings.” Furthermore, “CMR can be used for follow-up to assess aortic dilatation.” In aortic stenosis, “myocardial fibrosis is a major driver of LV decompensation < … > , which can be detected and quantified using CMR. Amyloidosis is also frequently associated with aortic stenosis in elderly patients < … > . When cardiac amyloidosis is clinically suspected, < … > scintigraphy and/or CMR should be considered.” In primary mitral regurgitation, “when various echocardiographic parameters used to grade mitral regurgitation are inconsistent, CMR is a valid alternative to quantify the regurgitant volume and is the reference standard to quantify left ventricular and left atrial volumes. In addition, quantification of mitral regurgitation with CMR has shown prognostic implications. Finally, preliminary data show that myocardial fibrosis assessed with CMR is frequent in primary mitral regurgitation and has been associated with sudden cardiac death and ventricular arrhythmias.” In asymptomatic patients with severe mitral regurgitation and normal LVEF, CMR is mentioned as a useful complementary diagnostic and risk stratification tool. In secondary mitral regurgitation, “the extent of myocardial scar, as assessed with CMR, has been associated with poor prognosis.” The use of 3D echocardiography, CMR and exercise echocardiography may help to identify patients with severe mitral regurgitation, when 2D echocardiography at rest is inconclusive. “In tricuspid regurgitation, CMR is the preferred method to assess the right ventricle due to its high accuracy and reproducibility. < … > Calculation of the tricuspid regurgitant volume by CMR using right ventricular volumetry may be helpful.”

##### CMR in specific recommendations

This guideline does not contain specific recommendation with referral to CMR.

##### Comparison between the current and the last guideline

The 2021 heart valve guideline contains similar statements regarding the role of CMR in the assessment of valvular heart disease compared to the versions from 2017 and 2012. In the 2021 guideline, the aspect of fibrosis imaging with CMR for risk stratification is slightly more pronounced.

#### 2021 ESC Guidelines on cardiovascular disease prevention in clinical practice [7]

##### CMR in specific recommendations

This guideline does not contain specific recommendation with referral to CMR.

##### CMR in the guideline text

CMR is mentioned in the context of the prevention of cardiotoxicity from tumor therapy using preventive medication. “The main benefits are less marked LV remodeling or a reduced decline in LVEF observed with CMR”. In patients with chronic obstructive pulmonary disease (COPD), in whom echocardiography, exercise tests and tests using vasodilators are limited, CMR is mentioned as important alternative to screen COPD patients for atherosclerotic cardiovascular disease.

##### Comparison between the current and the last guideline

The 2012 prevention guideline had concluded that CMR is a promising research tool, but its routine use remained limited, and it was not yet appropriate for identifying patients at high risk for cardiovascular disease. The 2016 prevention guideline did not mention CMR. The 2021 guideline revives CMR by mentioning it in the context of cardiotoxicity and risk assessment in patients with pulmonary disease.

#### 2021 ESC Guidelines on cardiac pacing and cardiac resynchronization therapy [8]

##### CMR in the guideline text

“In patients with suspected or documented symptomatic bradycardia, the use of cardiac imaging is recommended to evaluate the presence of structural heart disease, to determine LV systolic function, and to diagnose potential reversible causes of conduction disturbances. When coronary artery disease is suspected, coronary computed tomography (CT), angiography, or stress imaging is recommended. CMR and nuclear imaging techniques provide information on tissue characterization (inflammation, fibrosis/scar) and should be considered before pacemaker implantation when specific etiologies associated with conduction abnormalities are suspected (specially in young patients). LGE and T2 CMR techniques allow the diagnosis of specific causes of conduction disturbances (i.e. sarcoidosis and myocarditis). LGE CMR helps in the decision-making of individuals with arrhythmic events; the presence of large areas of LGE (scar/fibrosis) has been linked to an increased risk of ventricular arrhythmias regardless of LVEF and may indicate the need for an implantable cardioverter-defibrillator. T2 CMR sequences are suited for the detection of myocardial inflammation (i.e. edema and hyperemia) as a potential cause of transitory conduction abnormalities that may not need permanent pacemaker implantation.”

Regarding the “benefit of adding implantable cardioverter defibrillator in patients with indications for cardiac resynchronization therapy”, “further predictive power concerning the risk of ventricular arrhythmia may be derived by contrast-enhanced CMR-guided scar characterization.”

“Cardiac dyssynchrony, myocardial scar and site of latest activation of the LV in relation to the LV lead position have been associated with response to CRT. LVEF is the only parameter included in the guidelines for the selection of patients for CRT <…>. Echocardiography is the imaging technique of first choice for the assessment of LVEF. However, when intravenous contrast is not available and the acoustic window does not allow accurate assessment of LVEF, CMR or nuclear imaging should be considered. Strain imaging (based on echocardiography or CMR) to quantify LV systolic function has shown incremental prognostic value in heart failure and allows assessment of LV mechanical dyssynchrony. CMR with LGE techniques (which show the presence of myocardial scar tissue) provide the best resolution to differentiate ischemic cardiomyopathy and non-ischemic cardiomyopathy. The location (posterolateral) and extent (transmural vs. non-transmural and percentage of LV mass) of LGE on CMR or with nuclear techniques has been associated with the benefit from CRT.”

##### CMR in specific recommendations

This guideline contains 2 recommendations (1 × IC, 1 × IIa C) with referral to CMR (Table 7).

**Table 7:** Recommendations for CMR in the guideline on cardiac pacing and cardiac resynchronization therapy

Recommendation	Class	Level
*Recommendations regarding imaging before implantation*		
Cardiac imaging is recommended in patients with suspected or documented symptomatic bradycardia to evaluate the presence of structural heart disease, to determine LV systolic function, and to diagnose potential causes of conduction disturbances	I	C
Multimodality imaging (CMR, CT, PET) should be considered for myocardial tissue characterization in the diagnosis of specific pathologies associated with conduction abnormalities needing pacemaker implantation, particularly in patients younger than 60 years	IIa	C

*CMR*  cardiovascular magnetic resonance, *CT*  computed tomography, *PET*  positron emission tomography

##### Comparison between the current and the last guideline

The guideline from 2013 only contained a short note that CMR and other imaging techniques can be considered for patient selection to evaluate cardiac dyssynchrony, “However, the real value of these novel technologies remains to be determined”. In the 2021 guideline, CMR is pointed out much more to define the underlying cause for potential rhythm disturbances and to guide therapeutic decisions.

#### 2021 ESC Guidelines for the diagnosis and treatment of acute and chronic heart failure [9]

##### CMR in specific recommendations

This guideline contains 4 recommendations with referral to CMR: 2 × IC, 1 × IIaC, 1 × IIb B. (Table 8).

**Table 8:** Recommendations for CMR in the guidelines the diagnosis and treatment of acute and chronic heart failure

Recommendation	Class	Level
*Recommendations for specialized diagnostic tests for selected patients with chronic heart failure to detect reversible/treatable causes of heart failure*		
CMR is recommended for the assessment of myocardial structure and function in those with poor echocardiogram acoustic windows	I	C
CMR is recommended for the characterization of myocardial tissue in suspected infiltrative disease, Fabry disease, inflammatory disease (myocarditis), left ventricular non-compaction, amyloid, sarcoidosis, iron overload / haemochromatosis	I	C
CMR with LGE should be considered in dilated cardiomyopathy (DCM) to distinguish between ischemic and non-ischemic myocardial damage	IIa	C
Non-invasive stress imaging (CMR, stress echocardiography, SPECT, PET) may be considered for the assessment of myocardial ischemia and viability in patients with coronary artery disease (CAD) who are considered suitable for coronary revascularization	IIb	B

*CMR*  cardiovascular magnetic resonance, *LGE*  late Gadolinium enhancement, *SPECT*  single photon emission computed tomography, *PET*  positron emission tomography

##### CMR in the guideline text

To determine the underlying etiology of chronic heart failure, “CMR imaging with LGE, T1 mapping and extracellular volume will identify myocardial fibrosis/scar, which are typically subendocardial for patients with ischemic heart disease in contrast to the mid-wall scar typical of dilated cardiomyopathy. In addition, CMR allows myocardial characterization in, e.g. myocarditis, amyloidosis, sarcoidosis, Chagas disease, Fabry disease, LV non-compaction cardiomyopathy, haemochromatosis, and arrhythmogenic cardiomyopathy.” To differentiate the causes of heart failure, CMR is recommended in coronary artery disease, cardiomyopathies, congenital / infective / infiltrative heart disease, storage disorders, endomyocardial and pericardial disease.

Therapeutic decisions are often based on LVEF. “If assessment of EF is not possible by echocardiography, then CMR or rarely, nuclear techniques can be employed.” Regarding patient selection for ICD therapy, “tools to help risk stratification (e.g. scar burden on magnetic resonance imaging) can be helpful.” “Whether implantation of ICDs reduces mortality in those with an LVEF > 35% is unknown. There is an ongoing trial of ICD therapy in such patients with the presence of scar on CMR imaging.” In patients with new heart failure presenting during pregnancy or if there is diagnostic uncertainty, non-contrast CMR may be considered.

“Echocardiography is central for the diagnosis and monitoring of HCM, DCM, and arrhythmogenic cardiomyopathy (AC). CMR imaging provides more detailed morphological and prognostic information and should be performed at baseline.” “Initial diagnostic assessment in patients with suspected cardiomyopathy” includes “CMR imaging with T1 and T2 sequencing and LGE to visualize structural changes, storage, infiltration, inflammation, fibrosis and scarring.”

In dilated cardiomyopathy or hypokinetic non-dilated cardiomyopathy, “clinical evaluation, ECG, echocardiography and possibly CMR, must be performed in first-degree relatives of patients.” In suspected HCM, “increased LGE in a patchy mid-wall pattern in the most hypertrophied segment further suggest the presence of HCM.” The diagnosis of AC is “based upon the evaluation of a combination of the genetic factors < … > , documentation of ventricular arrhythmias and imaging criteria (echocardiography and MRI) of RV dysplasia with the fibrofatty replacement either or not confirmed by endomyocardial biopsy (EMB).” “Clinical evaluation, ECG, echocardiography and possibly CMR have to be performed in first-degree relatives who have the same definite disease-causing mutation as the index patient.” “ICD implantation should be considered for patients with DCM, HCM, or AC. The strength of the indication varies according to the clinical risk factors for sudden cardiac death with higher priority being given to those patients with significant LGE on CMR, younger age, or with a specific familial/genetic phenotype.” In atrial disease, “atrial size and function can be evaluated by multimodality imaging including two- and three-dimensional echocardiography, myocardial deformation, CT and CMR.”

Suspected acute myocarditis is defined as „clinical presentation +  ≥ 1 mandatory diagnostic test being positive (by preference CMR) in the absence of significant coronary artery, valvular or congenital heart disease, or other causes.” CMR is listed as mandatory test with high sensitivity and intermediate specificity. CMR provides “edema, inflammation and fibrosis detection, quantification and localization through T1 and T2 mapping, extracellular volume assessment and LGE.” If endomyocardial biopsy in patients with suspected myocarditis is performed, “CMR or PET guided sampling may be considered.” Table 33 of the guideline summarizes indication, main findings, and diagnostic significance of CMR in suspected myocarditis: “Indication: Indicated at baseline, in all patients with clinical history + ECG, elevated troponin or echocardiographic abnormalities, and significant CAD excluded or unlikely. Advised at follow-up in patients with persistent dysfunction at echocardiography, arrhythmias, or ECG abnormalities. Main findings: At baseline: T1-weighted (inflammation, injury) and T2-weighted (edema) sequences, extracellular volume and LGE within 2 weeks after symptom onset. At follow up: LGE to evaluate the degree of scarring, T1 and T2 to identify persistent inflammation. Diagnostic significance: At least one T2-based criterion (global or regional increase of myocardial T2 relaxation time or an increased signal intensity in T2-weighted images), with at least one T1-based criterion (increased myocardial T1, extracellular volume, or LGE) in the acute phase. Only one (i.e., T2-based or T1-based) marker may still support a diagnosis of acute myocardial inflammation in an appropriate clinical scenario, albeit with less specificity in the acute phase. A negative T1/T2 scan does not exclude a still ongoing inflammatory process in the chronic phase.”

In cardiac amyloidosis (CA), “cardiac imaging and EMB or extra-cardiac biopsy are needed for the diagnosis of AL-CA in patients with abnormal hematological tests.” In transthyretin (TTR) -CA, “CMR has a sensitivity and specificity of 85% and 92%.” Among the “red flags for most common forms of cardiac amyloidosis”, CMR is listed, with “subendocardial LGE, elevated native T1 values, increased extracellular volume and abnormal gadolinium kinetics” being present in AL-CA and TTR-CA.

In iron overload cardiomyopathy, “myocardial iron deposition can be accurately estimated by the CMR T2* technique; T2* values are correlated with left and right ventricular systolic function and predict the development of iron-induced HF or arrhythmias.”

##### Comparison between the current and the last guideline

Compared to the guideline version from 2016, the specific recommendations for CMR in heart failure remained unchanged. Compared to 2012, their number has increased from 2 to 4. Like previous guideline versions, CMR is recommended to clarify the cause of heart failure in various scenarios. The description of CMR is much more extensive and detailed in the 2021 guideline and regarding myocarditis, CMR is even rated as “mandatory”. The 2021 guideline attributes CMR a more pronounced role in the field of risk stratification in heart failure, especially based on LGE fibrosis imaging.

#### 2020 ESC Guidelines on sports cardiology and exercise in patients with cardiovascular disease [10]

##### CMR in specific recommendations

This guideline contains 5 recommendations with referral to CMR: 1 × I C, 1 × IIa B, 1 × IIaC, 1 × IIb C, 1 × III C (Table 9).

**Table 9:** Recommendations for CMR in the guidelines on sports cardiology and exercise in patients with cardiovascular disease

Recommendation	Class	Level
*Recommendations for exercise and participation in sports in individuals with aortic pathology*		
Prior to engaging in exercise, risk stratification, with careful assessment including advanced imaging of the aorta (CT/CMR) and exercise testing with blood pressure assessment is recommended	I	C
*Recommendations for exercise in individuals with left ventricular non-compaction cardiomyopathy (LVNC)*		
A diagnosis of LVNC in athletic individuals should be considered if they fulfill imaging criteria, in association with cardiac symptoms, family history of LVNC or cardiomyopathy, left ventricular systolic (EF < 50%) or diastolic (E’ < 9 cm/s) dysfunction, a thin compacted epicardial layer (< 5 mm in end-diastole on CMR, or < 8 mm in systole on echocardiography), or abnormal 12-lead ECG	IIa	B
*Recommendations for exercise in individuals with dilated cardiomyopathy (DCM)*		
Participation in high- or very high-intensity exercise including competitive sports (with the exception of those where occurrence of syncope may be associated with harm or death) may be considered in asymptomatic individuals who fulfill all of the following: (i) mildly reduced left ventricular systolic function (EF 45–50%); (ii) absence of frequent and/or complex ventricular arrhythmias on ambulatory Holter monitoring or exercise testing; (iii) absence of LGE on CMR; (iv) ability to increase EF by 10–15% during exercise; and (v) no evidence of high-risk genotype (lamin A/C or filamin C)	IIb	C
Participation in high- or very high-intensity exercise including competitive sports is not recommended for individuals with a DCM and any of the following: (i) symptoms or history of cardiac arrest or unexplained syncope; (ii) LVEF < 45%; (iii) frequent and/or complex ventricular arrhythmias on ambulatory Holter monitoring or exercise testing; (iv) extensive LGE (> 20%) on CMR; or (v) high-risk genotype (lamin A/C or filamin C)	III	C
*Recommendations for exercise in individuals with myocarditis*		
Return to all forms of exercise including competitive sports should be considered after 3–6 months in asymptomatic individuals, with normal troponin and biomarkers of inflammation, normal LV systolic function on echocardiography and CMR, no evidence of ongoing inflammation or myocardial fibrosis on CMR, good functional capacity, and absence of frequent and/or complex ventricular arrhythmias on ambulatory Holter monitoring or exercise testing	IIa	C

*CMR*  cardiovascular magnetic resonance, *CT*  computed tomography, *ECG*  electrocardiogram, *LGE*  late gadolinium enhancement, *LV*  left ventricular, *EF*  ejection fraction

##### CMR in the guideline text

In “Individuals at risk of atherosclerotic coronary artery disease and asymptomatic individuals in whom coronary artery disease is detected at screening”, “the increasing use of cardiac imaging techniques allows the identification of a greater number of individuals with asymptomatic chronic coronary syndrome, including competitive master athletes.”* “*Several methods of stress testing (e.g. cycle ergometry or treadmill testing), stress echocardiography, adenosine or dobutamine stress CMR, or positron emission tomography (PET)/single-photon emission computed tomography (SPECT), can be used to detect inducible myocardial ischemia.” “In the event of a borderline or uninterpretable exercise test result, it is recommended that a more specific imaging stress test is performed such as stress-echocardiography, CMR perfusion imaging, or SPECT.” Regarding “myocardial ischemia without obstructive disease in the epicardial coronary artery” (INOCA), “stress CMR and PET can detect abnormal coronary flow reserve and suggest coronary microvascular dysfunction with non-critical lesions.” In suspected anomalous origin of coronary arteries, “exercise testing rarely reveals myocardial ischemia and multislice contrast-enhanced CT, coronary CT angiography (CCTA), or CMR are the mainstay of diagnosis. In patients with aortic valve regurgitation, “in individuals with suboptimal echocardiographic images, CMR has the advantages of providing an accurate assessment of LV volume and EF, flow calculations and detecting the presence of myocardial scar in individuals with severe aortic regurgitation. Furthermore, the whole thoracic aorta can be visualized during the same examination.”

Individuals with mitral valve prolapse and “inferior T-wave inversion or ventricular premature beats arising from the LV should undergo a CMR imaging scan to check specifically for myocardial fibrosis affecting the infero-basal wall.” “The diagnosis of HCM is based on the presence of unexplained LV hypertrophy, defined as a maximum end-diastolic wall thickness ≥ 15 mm, in any myocardial segment on echocardiography, CMR, or CT imaging.” “CMR imaging is increasingly recognized as a necessary tool for confirming diagnosis and to assess risk stratification in individuals with HCM. LGE, indicative of myocardial fibrosis, may be present in up to 75% of patients with HCM and, by itself, is a poor discriminator of outcomes. However, the presence of extensive (≥ 15% of LV myocardium) LGE may identify individuals at increased risk of ventricular tachyarrhythmias and sudden cardiac death.” In arrhythmogenic cardiomyopathy, “in relation to risk stratification for sudden cardiac death, the clinician should assess the severity of RV and LV involvement in terms of ventricular dilatation and systolic dysfunction. CMR imaging is more useful than echocardiography for assessing RV wall motion abnormalities and can also quantify the degree of myocardial fat infiltration and/or scar.” Athletes with suspected left ventricular non-compaction, “will require further assessment with CMR, exercise echocardiography, and Holter monitor to assess the presence of LV fibrosis, cardiac thrombi, contractile reserve, and exercise-induced complex arrhythmias.” “In individuals with DCM, CMR has emerged as an important tool for the diagnosis and risk stratification of DCM. Specifically, the presence of LGE, with the typical mid-wall distribution, has been associated with increased risk of ventricular arrhythmias and sudden cardiac death.” Regarding myocarditis, “CMR is the most useful diagnostic tool and has excellent sensitivity for detecting myocardial hyperemia, inflammation, oedema and/or focal scar. The Lake Louise Criteria and LGE are now complemented by CMR techniques of T1/T2 mapping and extracellular volume fraction (ECV). The extent and distribution of LGE with non-ischemic pattern are independent predictors of cardiovascular events during follow-up. Namely, a 10% increase of LGE volume conveys a 79% increase in the risk of major cardiovascular events.” “Both the ESC and AHA recommend abstinence from moderate- to high-intensity exercise for a period of 3–6 months, although the precise timing for return to competitive or recreational sports involving moderate- or high-intensity exercise may be guided by the presence of inflammation on T2-weighted images and LGE uptake on CMR.” “Individuals with myocarditis should have a comprehensive evaluation after complete recovery to assess the risk of exercise-related sudden cardiac death. Imaging studies, exercise stress test, and Holter monitor provide essential information for risk stratification. Depressed LV function, presence of LGE and complex ventricular arrhythmias during exercise or Holter monitoring are recognized risk markers for adverse outcomes. Repeat evaluation should consist of measurement of troponin and biomarkers of inflammation, echocardiography, and prolonged ECG monitoring. < … > A CMR should be repeated if myocardial oedema or LGE was present during the acute illness. Return to sporting activities should be considered, in asymptomatic individuals, with normal troponin and biomarkers of inflammation, normal LV systolic function on echocardiography and CMR, no evidence of ongoing inflammation or myocardial fibrosis on CMR, good functional capacity, and absence of complex arrhythmias during exercise on prolonged ECG monitoring. Individuals with previous myocarditis are at risk of recurrence and silent clinical progression, and the presence of LGE during the acute presentation is associated with increased incidence of major adverse cardiac events; therefore, periodic re-evaluation is advised on an annual basis. Among individuals with healed myocarditis with persistence of LGE on CMR but no myocardial oedema at 3–6 months, those who are asymptomatic, with normal troponin and biomarkers of inflammation, normal LV systolic function, no evidence of ongoing inflammation on CMR, and absence of complex arrhythmias during exercise on prolonged ECG monitoring (48 h Holter ECG and exercise stress testing), should be evaluated on a case by case basis and may return to competitive sports on an individual basis. In contrast, individuals with extensive myocardial scar (> 20% LGE) and persistent LV dysfunction should abstain from exercise programs and sports activities involving moderate or high physical intensity.” In suspected pericarditis, “CMR should be considered in individuals with raised cardiac troponin levels to assess for concomitant myocardial inflammation. Furthermore, CMR will identify active inflammation of the pericardium, thickened pericardial layers, and any signs of pericardial constriction.”

“Premature ventricular contractions induced by exercise should be considered as a ‘red flag’, because ventricular arrhythmias associated with heart diseases are often made worse by adrenergic stimulation. A higher prevalence of myocardial substrates (mainly mid-wall or subepicardial non-ischemic LV scars) was found in a CMR study among athletes with exercise-induced PVCs compared to those with exercise-suppressed ventricular arrhythmias.”

For “assessment of the athlete with congenital heart disease”, “CMR scanning may be a preferable modality in complex disease. This has the additional benefit of evaluating intracardiac scar, which may inform the assessment of arrhythmia risk.” Finally, aortic size can be measured “usually by echocardiography or CMR, coarctation should be excluded.”

##### Comparison between the current and the last guideline

This guideline from 2021 is the first one with this topic. A comparison to previous versions is not possible.

#### 2020 ESC Guidelines for the management of acute coronary syndromes in patients presenting without persistent ST-segment elevation [11]

##### CMR in specific recommendations

This guideline contains 1 recommendation (I B) with referral to CMR (Table 10).

**Table 10:** Recommendations for CMR in guidelines for the management of acute coronary syndromes in patients presenting without persistent ST-segment elevation

Recommendation	Class	Level
*Recommendations for myocardial infarction with non-obstructive coronary arteries (MINOCA)*		
It is recommended to perform CMR in all MINOCA patients without an obvious underlying cause	I	B

*CMR*  cardiovascular magnetic resonance

##### CMR in the guideline text

“CMR can assess both perfusion and wall motion abnormalities, and patients presenting with acute chest pain with a normal stress CMR have an excellent short- and mid-term prognosis. Additionally, CMR permits detection of scar tissue (using late gadolinium enhancement) and can differentiate this from recent infarction (using T2-weighted imaging to delineate myocardial oedema). Moreover, CMR can facilitate the differential diagnosis between infarction, myocarditis, or Takotsubo syndrome, among others.”

“Most of the ‘rule-in’ patients with diagnoses other than myocardial infarction did have conditions that usually still require invasive coronary angiography or CMR imaging for accurate diagnosis, including Takotsubo syndrome and myocarditis.” “Even after the rule-out of myocardial infarction, elective non-invasive or invasive imaging may be indicated according to clinical assessment.” “Stress imaging by CMR, stress echocardiography, or nuclear imaging may also be an option based on risk assessment.” “Patients who do not qualify for ‘rule-out’ or ‘rule-in’, are assigned to observe.” “In patients with low-to-intermediate likelihood for this condition according to clinical judgment, non-invasive imaging using CCTA or stress testing [stress echocardiography, PET, SPECT, or CMR for the detection of acute coronary syndrome features (oedema, late gadolinium enhancement, perfusion defect, etc.)] should be considered after discharge from the emergency department to the ward.” Patients with NSTEMI-ACS diagnosis and “with no recurrence of symptoms and none of the very high or high-risk criteria listed in the recommendation table regarding timing of invasive strategy are to be considered at low risk of short-term acute ischaemic events. <…> In this setting, stress echocardiography or stress CMR may be preferred over non-invasive anatomical testing.”

In suspected myocardial infarction with non-obstructive coronary arteries (MINOCA), CMR is one of the key diagnostic tools < … > for the differential diagnosis of Takotsubo syndrome, myocarditis, or true myocardial infarction. CMR has the ability to identify the underlying cause in as many as 87% of patients with MINOCA. In the sub-endocardium, late gadolinium enhancement may indicate an ischemic cause, while sub-epicardial localization may indicate cardiomyopathies or myocarditis, and the absence of relevant late gadolinium enhancement with oedema and associated specific wall motion abnormalities is a hallmark of Takotsubo syndrome.”

##### Comparison between the current and the last guideline

Compared to the last guideline version from 2015, the extent of text passages describing the use of CMR has increased in the 2020 version. In particular, the role of CMR in MINOCA has been pronounced and led to the first specific recommendation for CMR in this context.

#### 2020 ESC Guidelines for the diagnosis and management of atrial fibrillation [12]

##### CMR in specific recommendations

This guideline does not contain specific recommendations with referral to CMR.

##### CMR in the guideline text

Imaging in atrial fibrillation by CMR provides information about left atrial anatomy, function, structure and thrombus detection. “Anatomical imaging provides the left atrial size, shape, and fibrosis. Most accurate assessment of left atrial dilation is obtained by CMR or CT.” “Assessment of left atrial fibrosis with LGE-CMR has been described but only rarely applied in clinical practice.” LGE CMR of the left atrium is recommended in selected patients to help decision making in atrial fibrillation treatment. “LA wall infiltration by epicardial fat is a potential early marker of inflammation and can be detected with CT or cardiac MRI. Before atrial fibrillation ablation, the pulmonary vein anatomy can be visualized with CT or CMR.” “Diagnostic work-up and follow-up in atrial fibrillation patients”, includes ischemia imaging in patients with suspected coronary artery disease.

##### Comparison between the current and the last guidelines

CMR is presented slightly more detailed in the 2020 guideline compared to the precursor from 2016. Back in 2012, the guideline about atrial fibrillation had not mentioned CMR at all.

#### 2020 ESC Guidelines for the management of adult congenital heart disease [13]

##### CMR in specific recommendations

This guideline contains 3 recommendations with referral to CMR (Table 11): 1 × I C and 2 × IIa C.

**Table 11:** Recommendations for CMR in the guidelines for the management of adult congenital heart disease

Recommendation	Class	Level
*Recommendations for intervention after repair of tetralogy of Fallot (TOF)*		
Electrophysiologic evaluation, including programmed electrical stimulation, should be considered for risk stratification for sudden cardiac death (SCD) in patients with additional risk factors (LV/RV dysfunction; non-sustained, symptomatic ventricular tachycardia (VT); QRS duration ≥ 180 ms, extensive RV scarring on CMR)	IIa	C
ICD implantation should be considered in selected TOF patients with multiple risk factors for SCD, including LV dysfunction, non-sustained, symptomatic VT, QRS duration ≥ 180 ms, extensive RV scarring on CMR, or inducible VT at programmed electrical stimulation	IIa	C
*Recommendations for the management of patients with anomalous coronary arteries*		
Non-pharmacological functional imaging (e.g. nuclear study, echocardiography, or CMR with physical stress) is recommended in patients with coronary anomalies to confirm/exclude myocardial ischemia	I	C

*CMR*  cardiovascular magnetic resonance, *LV*  left ventricular, *RV*  right ventricular, *ICD*  implantable cardioverter defibrillator

##### CMR in the guideline text

In adult congenital heart disease (ACHD), “noninvasive imaging is routinely performed by transthoracic echocardiography involving transesophageal echocardiography and CMR imaging where indicated.” “CMR is ideal for accurate quantification of ventricular volumes, ejection fraction, valvular regurgitation, calculation of pulmonary and systemic blood flow, and myocardial fibrosis assessment.”

“CMR has become an essential facility in the specialist unit. It enables 3D anatomical reconstruction, which is not restricted by body size or acoustic windows and has rapidly improving spatial and temporal resolution. <…> CMR is the gold-standard imaging method for quantification of volumes. It may be an alternative when echocardiography cannot be obtained with sufficient quality or used as a second method when echocardiography measurements are borderline or ambiguous. Furthermore, the lack of radiation makes it a useful tool when serial evaluations are needed (e.g. for monitoring aortic dimensions). CMR allows calculation of systemic and pulmonary blood flow in patients with multiple sources of blood supply and, in combination with invasive catheterization, of pulmonary vascular resistance. Tissue characterization for myocardial fibrosis is a unique capability of CMR. Late gadolinium enhancement CMR for focal fibrosis and interstitial fibrosis T1 mapping imaging are increasingly being applied in ACHD for their potential diagnostic and prognostic value. However, large CHD lesion-specific studies to determine if they predict survival are ongoing. Adults with CHD with conventional pacemakers and defibrillators can undergo CMR within guidelines where local support is available. 3D CMR imaging can be integrated into electrophysiology procedures to inform and guide them. 3D CCT and CMR reconstructions can also be used for virtual reality rehearsal of interventions or planning from patient-specific 3D prints.”

Indications for CMR in ACHD are given in Table 6 of the original guideline: “Quantification of RV volumes, EF (including subpulmonary RV, systemic RV, and single ventricle). Evaluation of RVOT-obstruction and RV-PA conduits. Quantification of pulmonary regurgitation. Evaluation of pulmonary arteries (stenoses, aneurysms) and the aorta [aneurysm, dissection, coarctation). Evaluation of systemic and pulmonary veins (anomalous connection, obstruction, coronary venous anatomy pre-procedure, etc.). Collaterals and arteriovenous malformations. Coronary anomalies and coronary artery disease. Detection and quantification of myocardial ischemia by CMR stress perfusion. Evaluation of intra- and extracardiac masses. Quantification of myocardial mass (LV and RV). Detection and quantification of myocardial fibrosis/scar (late gadolinium enhancement, T1 mapping) tissue characterization (fibrosis, fat, iron, etc.). Quantification of systemic and pulmonary blood flow to calculate Qp:Qs. Quantification of perfusion distribution to the right/left lung. Measurement of pulmonary blood flow in patients with multiple sources of blood supply (i.e. with major aorto-pulmonary collateral arteries).”

In the subsequent guideline text, various specific congenital lesions are described separately. CMR is mentioned for diagnostic work-up or follow-up in all of them. For tetralogy of Fallot and for coronary anomaly, specific recommendations are given (Table 11). The content of these paragraphs overlaps with the general description of CMR in ACHD. Details are given in the Additional file 1.

##### Comparison between the current and the last guideline

Structure and content regarding CMR remained widely constant between the guideline versions from 2020 and 2010. New in 2020 compared to 2010 are the specific recommendations regarding risk stratification in patients with tetralogy of Fallot based on scar assessment of the right ventricle using CMR.

#### 2019 ESC Guidelines for the diagnosis and management of chronic coronary syndromes [14]

##### CMR in specific recommendations

This guideline contains 10 recommendations with referral to CMR (Table 12): 4 × I B, 1 × I C, 2 × IIb B, 2 × IIb C, 1 × III C.

**Table 12:** Recommendations for CMR in the guidelines for the diagnosis and management of chronic coronary syndromes

Recommendation	Class	Level
*CMR in the initial diagnostic management of patients with suspected coronary artery disease (CAD)*		
CMR may be considered in patients with an inconclusive echocardiographic test	IIb	C
*Use of diagnostic imaging tests in the initial diagnostic management of symptomatic patients with suspected CAD*		
Non-invasive functional imaging for myocardial ischemia or coronary CTA is recommended as the initial test for diagnosing CAD in symptomatic patients in whom obstructive CAD cannot be excluded by clinical assessment alone	I	B
It is recommended that selection of the initial non-invasive diagnostic test is done based on the clinical likelihood of CAD and other patient characteristics that influence test performance, local expertise, and the availability of tests	I	C
Functional imaging for myocardial ischemia is recommended if coronary CTA has shown CAD of uncertain functional significance or is not diagnostic	I	B
*Recommendations on risk assessment*		
Risk stratification, preferably using stress imaging or coronary CTA (if local expertise and availability permit), or alternatively exercise stress ECG (if significant exercise can be performed and the ECG is amenable to the identification of ischemic changes), is recommended in patients with suspected or newly diagnosed CAD	I	B
*Recommendations for screening for coronary artery disease in asymptomatic subjects*		
In high-risk asymptomatic adults (with diabetes, a strong family history of CAD, or when previous risk-assessment tests suggest a high risk of CAD), functional imaging or coronary CTA may be considered for cardiovascular risk assessment	IIb	C
In low-risk non-diabetic asymptomatic adults, coronary CTA or functional imaging for ischemia is not indicated for further diagnostic assessment	III	C
In asymptomatic adults (age > 40 years) with diabetes, functional imaging or coronary CTA may be considered for advanced cardiovascular risk assessment	IIb	B
*Recommendations for symptomatic patients with a long-standing diagnosis of chronic coronary syndromes*		
Risk stratification is recommended in patients with new or worsening symptom levels, preferably using stress imaging or, alternatively, exercise stress ECG	I	B
*Investigations in patients with suspected coronary microvascular angina*		
Transthoracic Doppler of the LAD, CMR, and PET may be considered for non-invasive assessment of coronary flow reserve (CFR)	IIb	B

*CMR*  cardiovascular magnetic resonance, *CTA*  computed tomography angiography, *ECG*  electrocardiogram, *LAD*  left anterior descending coronary artery, *PET*  positron emission tomography

##### CMR in the guideline text

“CMR may be considered in patients with suspected CAD when the echocardiogram (having used contrast) is inconclusive. CMR will provide useful information on cardiac anatomy and systolic cardiac function, similar to that from an echocardiogram <…>. CMR can assess global and regional function, and the use of late gadolinium enhancement CMR can reveal a typical pattern of scarred myocardium in patients who have already experienced an MI.” Using CMR for functional stress testing, “≥2 of 16 segments with stress perfusion defects or ≥3 dobutamine-induced dysfunctional segments” are indicators of high event risk in patients with established chronic coronary syndromes.”

“If the diagnosis of CAD is uncertain, establishing a diagnosis using non-invasive functional imaging for myocardial ischemia before treatment is reasonable.” “The current Guidelines recommend the use of either noninvasive functional imaging of ischemia or anatomical imaging using coronary CT angiography (CTA) as the initial test for diagnosing CAD.” “Functional non-invasive tests for the diagnosis of obstructive CAD are designed to detect myocardial ischemia through ECG changes, wall motion abnormalities by stress CMR or stress echocardiography, or perfusion changes by single-photon emission CT (SPECT), positron emission tomography (PET), myocardial contrast echocardiography, or contrast CMR.”

“The non-invasive functional tests for ischemia typically have better rule-in power. In outcome trials, functional imaging tests have been associated with fewer referrals for downstream invasive coronary angiography (ICA) compared with a strategy relying on anatomical imaging. Before revascularization decisions can be made, functional evaluation of ischemia (either non-invasive or invasive) is required in most patients. Therefore, functional non-invasive testing may be preferred in patients at the higher end of the range of clinical likelihood if revascularization is likely or the patient has previously diagnosed CAD.”

When “anatomical non-invasive evaluation” is used, “either non-invasive or invasive functional testing is recommended for further evaluation of angiographic stenosis detected by coronary CTA or invasive angiography, unless a very high-grade (> 90% diameter stenosis) stenosis is detected via invasive angiography.”

##### Comparison between the current and the last guideline

The 2019 guideline about chronic coronary syndrome replaced the 2013 guideline about stable coronary artery disease. The position of non-invasive testing for coronary artery disease has been strengthened, with a more detailed differentiation between anatomic and functional testing. The various functional tests (SPECT, CMR, stress echocardiography) are regarded as widely equally potent for that purpose. The structure and formulation of the specific recommendations has changed without significant change in the content.

#### 2019 ESC Guidelines on diabetes, pre-diabetes, and cardiovascular diseases developed in collaboration with the EASD [15]

##### CMR in specific recommendations

This guideline contains 3 recommendations with referral to CMR (Table 13): 1 × I C and 2 × IIb B.

**Table 13:** Recommendations for CMR in the guidelines on diabetes, pre-diabetes, and cardiovascular diseases

Recommendation	Class	Level
*Recommendations for the use of imaging testing for cardiovascular risk assessment in asymptomatic patients with diabetes*		
CT coronary angiography (CTCA) or functional imaging (radionuclide myocardial perfusion imaging, stress CMR imaging, or exercise or pharmacological stress echocardiography) may be considered in asymptomatic patients with diabetes mellitus (DM) for screening of coronary artery disease (CAD)	IIb	B
Detection of atherosclerotic plaque of carotid or femoral arteries by CT, or magnetic resonance imaging, may be considered as a risk modifier in patients with DM at moderate or high risk	IIb	B
*Recommendations for the diagnosis and management of peripheral arterial disease in patients with diabetes*		
CT angiography or magnetic resonance angiography is indicated in case of lower extremity arterial disease when revascularization is considered	I	C

*CMR*  cardiovascular magnetic resonance, *CT*  computed tomography

##### CMR in the guideline text

“CMR and tissue characterization techniques have shown that patients with diabetes mellitus (DM) without CAD have diffuse myocardial fibrosis as the mechanism of LV systolic and diastolic dysfunction.” “Stress testing or CTCA may be indicated in very high-risk asymptomatic individuals (with peripheral arterial disease, a high coronary artery calcium (CAC) score, proteinuria, or renal failure)”. “Stress testing with myocardial perfusion imaging or stress echocardiography permits the detection of silent myocardial ischemia.” “In patients with DM with frequent symptomatic premature ventricular beats or episodes of non-sustained VT, the presence of underlying structural heart disease should be examined by exercise ECG, echocardiography, coronary angiography, or magnetic resonance imaging.”

##### Comparison between the current and the last guideline

Compared to the 2013 version, the current guideline newly includes stress CMR for functional imaging in diabetes and suspected CAD. Whereas the 2013 did not contain specific recommendations with regard to CMR, the 2019 version includes three recommendations.

#### 2019 ESC/EAS Guidelines for the management of dyslipidaemias: lipid modification to reduce cardiovascular risk [16]

Unchanged to the guideline from 2010 and 2016, the 2019 version does not mention CMR in the text and does not contain recommendations with referral to CMR.

#### 2019 ESC Guidelines for the diagnosis and management of acute pulmonary embolism [17]

##### CMR in specific recommendations

This guideline contains 1 recommendation (III A) with referral to CMR (Table 14).

**Table 14:** Recommendations for CMR in guidelines for the diagnosis and management of acute pulmonary embolism

Recommendation	Class	Level
***Recommendations for diagnosis***		
Magnetic resonance angiography is not recommended for ruling out pulmonary embolism	III	A

*CMR*  cardiovascular magnetic resonance

##### CMR in the guideline text

“Magnetic resonance angiography (MRA) has been evaluated for several years regarding suspected PE. However, the results of large-scale studies show that this technique, although promising, is not yet ready for clinical practice due to its low sensitivity, the high proportion of inconclusive MRA scans, and its low availability in most emergency settings.”

##### Comparison between the current and the last guideline

There is no change regarding CMR between the 2019 guideline and the version from 2014.

#### 2019 ESC Guidelines for the management of patients with supraventricular tachycardia [18]

##### CMR in specific recommendations

This guideline does not contain recommendations with referral to CMR.

##### CMR in the guideline text

“In patients with suspected tachycardia induced cardiomyopathy, CMR is advisable to exclude intrinsic structural change.” Regarding ‘further research’, CMR is mentioned as a potential surrounding for a radiation-free electrophysiology laboratory.

##### Comparison between the current and the last guideline

This is the first guideline with this topic.

#### 2018 ESC/EACTS Guidelines on myocardial revascularization [19]

##### CMR in specific recommendations

This guideline contains 4 recommendations with referral to CMR (Table 15). 1 × IIa B, 1 × IIb B, 2 × IIb C.

**Table 15:** Recommendations for CMR in the guideline on myocardial revascularization

Recommendation	Class	Level
*Recommendations for non-invasive imaging in patients with coronary artery disease (CAD) and heart failure with reduced ejection fraction*		
Non-invasive stress imaging (CMR, stress echocardiography, SPECT, or PET) may be considered for the assessment of myocardial ischemia and viability in patients with heart failure and CAD (considered suitable for coronary revascularization) before the decision on revascularization	IIb	B
*Strategies for follow-up and management in symptomatic patients after myocardial revascularization*		
An imaging stress test should be considered in patients with prior revascularization over stress ECG	IIa	B
*Strategies for follow-up and management in asymptomatic patients after myocardial revascularization*		
Surveillance by non-invasive imaging-based stress testing may be considered in high-risk patient subsets 6 months after revascularization	IIb	C
Routine non-invasive imaging-based stress testing may be considered 1 year after PCI and > 5 years after CABG	IIb	C

*CMR*  cardiovascular magnetic resonance, *SPECT*  single photon emission computed tomography, *PET*  positron emission tomography, *ECG*  electrocardiogram, *PCI*  percutaneous coronary intervention, *CABG*  coronary artery bypass grafting

##### CMR in the guideline text

“Non-invasive diagnostic assessment of patients with CAD being considered for myocardial revascularization comprises the assessment of ischemia and the evaluation of viability in patients with regional wall motion abnormalities or reduced ejection fraction (EF). Functional testing to assess ischemia is critical for the assessment of stable patients with CAD. Documentation of ischemia using functional testing before elective invasive procedures for CAD is the preferred approach. <…> Because of the low sensitivity of exercise electrocardiogram (ECG) testing in the assessment of patients with symptoms of angina, non-invasive imaging is recommended as the first-line test. Detection of a large area of myocardial ischemia by functional imaging is associated with impaired prognosis of patients and identifies patients who should undergo revascularization.”

“Assessment of myocardial viability may be done in order to select patients that are more likely to benefit from myocardial revascularization and can be achieved with several imaging modalities: myocardial contrast echocardiography, single-photon emission CT (SPECT), and late gadolinium enhancement cardiac magnetic resonance (LGE-CMR) all assess cellular integrity; positron emission tomography (PET) assesses cellular metabolism; and dobutamine techniques assess contractile reserve. Assessment of ischemia provides incremental benefit over viability in mild to moderate CAD, but with extensive CAD viability assessment may be sufficient.”

##### Comparison between the current and the last guideline

The current guideline from 2018 is widely constant to the version from 2014. Again, CMR is mainly recommended for ischemia testing. The role of viability testing by CMR and other techniques is still mentioned without particular emphasis, as the evidence is still controversial.

#### 2018 ESC/ESH Guidelines for the management of arterial hypertension [20]

##### CMR in specific recommendations

This guideline does not contain specific recommendations with referral to CMR.

##### CMR in the guideline text

CMR is mentioned as “the gold standard for cardiac anatomical and functional quantification”. For the evaluation of left ventricular hypertrophy as a marker of hypertension-mediated organ damage, CMR is evaluated with “high sensitivity” (superior to ECG and echocardiography) and with “high reproducibility and operator independence”.

##### Comparison between the current and the last guideline

In contrary to the 2014 version, the 2018 guideline does not contain a specific recommendation for stress imaging including CMR in suspected CAD and the option for CMR in renal artery stenosis.

#### 2018 ESC Guidelines for the management of cardiovascular diseases during pregnancy [21]

##### CMR in specific recommendations

This guideline contains 3 recommendations with referral to CMR (Table 16): 2 × I C, 1 × IIa C.

**Table 16:** Recommendations for CMR in guideline for the management of cardiovascular diseases during pregnancy

Recommendation	Class	Level
*General recommendations*		
MRI (without gadolinium) should be considered if echocardiography is insufficient for a definite diagnosis	IIa	C
*Recommendations for the management of aortic disease*		
Imaging of the entire aorta (CT/MRI) is recommended before pregnancy in patients with a genetically proven aortic syndrome or known aortic disease	I	C
For imaging of pregnant women with dilatation of the distal ascending aorta, aortic arch, or descending aorta, MRI (without gadolinium) is recommended	I	C

*CMR*  cardiovascular magnetic resonance, *MRI*  magnetic resonance imaging, *CT*  computed tomography

##### CMR in the guideline text

“All women with known cardiac or aortic disease who wish to embark on pregnancy require timely pre-pregnancy counselling.” “In case of aortic pathology, complete aortic imaging by CT scanning or MRI is necessary for appropriate pre-conception counselling.” “MRI is advised if other non-invasive diagnostic measures are not sufficient for definitive diagnosis and is preferred to ionizing radiation based imaging modalities when possible.” “Depending on the aortic diameter, patients with aortic pathology should be monitored by echocardiography at regular intervals throughout the pregnancy and 6 months post-partum. <…> When needed, cardiac MRI without contrast can be used.” In bicuspid valve disease, “aortic dilatation <…> can occur even when valve function is normal. The dilatation can be in the distal ascending aorta, which cannot be adequately visualized by echocardiography. If not visible with echocardiography, MRI or CT should be performed pre-pregnancy.”

##### Comparison between the current and the last guideline

The content regarding CMR remained widely constant between the 2018 and the 2011 guideline versions.

#### 2018 ESC Guidelines for the diagnosis and management of syncope [22]

##### CMR in specific recommendations

This guideline does not contain specific recommendations with referral to CMR.

##### CMR in the guideline text

“Computed tomography or CMR should be considered in selected patients presenting with syncope of suspected cardiac structural origin when echocardiography is not diagnostic.” In arrhythmogenic right ventricular cardiomyopathy, unexplained syncope may be a marker of arrhythmic risk in patients and the presence of LGE on CMR may influence the decision to implant an ICD. ‘

##### Comparison between the current and the last guideline

Compared to the guideline version from 2009, the 2018 guideline newly contains statements about CMR for structural heart assessment, concretely in the case of arrhythmogenic heart disease.

#### 2017 ESC Guidelines on the diagnosis and treatment of peripheral arterial diseases [23]

##### CMR in specific recommendations

This guideline contains 5 recommendations with referral to CMR (Table 17): 4 × I B, 1 × I C.

**Table 17:** Recommendations for CMR in the guidelines on the diagnosis and treatment of peripheral arterial diseases

Recommendation	Class	Level
*Recommendations for imaging of extracranial carotid arteries*		
Duplex ultrasound (as first-line imaging), CTA and/or MRA are recommended for evaluating the extent and severity of extracranial carotid stenoses	I	B
When carotid artery stenosis (CAS) is being considered, it is recommended that any duplex ultrasound study be followed by either MRA or CTA to evaluate the aortic arch as well as the extra- and intracranial circulation	I	B
When carotid endarterectomy (CEA) is considered, it is recommended that the duplex ultrasound stenosis estimation be corroborated by either MRA or CTA (or by a repeat duplex ultrasound study performed in an expert vascular laboratory)	I	B
*Recommendations for diagnostic strategies for renal artery disease*		
Duplex ultrasound (as first-line), CTA and MRA are recommended imaging modalities to establish a diagnosis of renal artery disease	I	B
*Recommendations on imaging in patients with lower extremity artery disease*		
Duplex ultrasound and/or CTA and/or MRA are indicated for anatomical characterization of lower extremity artery disease lesions and guidance for optimal revascularization strategy	I	C

*CMR*  cardiovascular magnetic resonance, *CTA*  computed tomography angiography, *MRA*  magnetic resonance angiography

##### CMR in the guideline text

Magnetic resonance angiography (MRA) “is used for peripheral artery imaging using contrast (i.e. gadolinium) and non-contrast techniques (i.e. phase contrast and time-of flight sequences). < … > Compared with CTA, MRA does not need iodine contrast and has higher soft tissue resolution; however, motion artefacts are more frequent.” “In a metaanalysis, duplex ultrasound, MRA and CTA were equivalent for detecting significant carotid stenosis.” In vertebral artery disease, “CTA/MRA have a higher sensitivity and specificity than duplex ultrasound.” In extracranial carotid and vertebral disease, “plaque morphological evaluation using MRI < … > may identify patients with asymptomatic stenoses at higher risk of ipsilateral ischemic stroke.” In upper extremity artery disease, CMR “provides both functional and morphological information useful to distinguish anterograde from retrograde perfusion and to estimate stenosis severity.” In renal artery disease, “Gadolinium-enhanced MRA provides excellent characterization of renal arteries, the surrounding vessels, renal mass and even renal excretion function.”

##### Comparison between the current and the last guideline

Regarding the role of CMR, the guideline from 2017 remained widely unchanged compared to the version from 2011.

#### 2017 ESC Guidelines for the management of acute myocardial infarction in patients presenting with ST-segment elevation [24]

##### CMR in specific recommendations

This guideline contains 3 recommendations with referral to CMR (Table 18): 2 × IIa C, 1 × IIb C.

**Table 18:** Recommendations for CMR in the guidelines for the management of acute myocardial infarction in patients presenting with ST-segment elevation (STEMI)

Recommendation	Class	Level
*Indications for imaging and stress testing in STEMI patients: during hospital stay (after primary PCI)*		
When echocardiography is suboptimal/inconclusive, an alternative imaging method (CMR preferably) should be considered	IIa	C
Either stress echo, CMR, SPECT, or PET may be used to assess myocardial ischemia and viability, including in multivessel CAD	IIb	C
*Indications for imaging and stress testing in STEMI patients: after discharge*		
When echo is suboptimal or inconclusive, alternative imaging methods (CMR preferably) should be considered to assess LV function	IIa	C

*CMR* cardiovascular magnetic resonance, *PCI* percutaneous coronary intervention, *SPECT* single photon emission computed tomography, *PET* positron emission tomography, *LV* left ventricular

##### CMR in the guideline text

In patients with ST-elevated myocardial infarction (STEMI), LGE CMR is named “the current state of the art for microvascular obstruction (MVO) identification and quantification. After STEMI, CMR is recommended for evaluating the cardiac state and for risk assessment by assessing myocardial perfusion and viability. “LGE-CMR imaging has a high diagnostic accuracy for assessing the transmural extent of myocardial scar tissue. However, the ability to detect viability and predict recovery of wall motion is not significantly superior to other imaging techniques. The presence of dysfunctional viable myocardium by LGE CMR is an independent predictor of mortality in patients with ischemic LV dysfunction.” For risk estimation in post-infarct patients with reduced LV-EF, additional parameters are named, predominantly obtained with CMR: infarct size, myocardium at risk, microvascular obstruction, intramyocardial hemorrhage.

In suspected myocardial infarction with non-obstructive coronary arteries (MINOCA), “CMR is a very helpful imaging technique due to its unique noninvasive tissue characterization, allowing the identification of wall motion abnormalities, presence of oedema, and myocardial scar/fibrosis presence and pattern. Performance of CMR within 2 weeks after onset of symptoms should be considered to increase the diagnostic accuracy of the test for identifying the etiological cause of MINOCA.”

##### Comparison between the current and the last guideline

Compared to the previous version from 2011, the 2017 guideline newly emphasizes the concept of MINOCA and the value of CMR for determining the final diagnosis and newly introduces various tissue parameters obtained from CMR for risk estimation in STEMI patients with persistent LV dysfunction.

#### 2015 ESC Guidelines for the diagnosis and management of pericardial diseases [25]

##### CMR in specific recommendations

This guideline contains 5 recommendations with referral to CMR: 3 × I C, 1 × IIa C, 1 × IIb C (Table 19).

**Table 19:** Recommendations for CMR in the guidelines for the diagnosis and management of pericardial diseases

Recommendation	Class	Level
*Recommendations for the diagnosis and management of pericarditis associated with myocarditis*		
Cardiac magnetic resonance is recommended for the confirmation of myocardial involvement	I	C
*Recommendations for the diagnosis of pericardial effusion*		
CT or CMR should be considered in suspected cases of loculated pericardial effusion, pericardial thickening, and masses, as well as associated chest abnormalities	IIa	C
*Recommendations for the diagnosis of constrictive pericarditis*		
CT and/or CMR are indicated as second-level imaging techniques to assess calcifications (CT), pericardial thickness, degree, and extension of pericardial involvement	I	C
*Recommendations for therapy of constrictive pericarditis*		
Empiric anti-inflammatory therapy may be considered in cases with transient or new diagnosis of constriction with concomitant evidence of pericardial inflammation (i.e. CRP elevation or pericardial enhancement on CT/CMR)	IIb	C
*Recommendations for the general diagnostic work-up of pericardial diseases*		
CT and/or CMR are recommended as second-level testing for diagnostic workup in pericarditis	I	C

*CMR*  cardiovascular magnetic resonance, *CT*  computed tomography, *CRP*  C-reactive protein

##### CMR in the guideline text

CMR allows “visualization and tissue characterization of the pericardium (and heart) in patients with pericardial disease and appraisal of the consequences of pericardial abnormalities on cardiac function and filling patterns. As such, it is probably the preferred imaging modality to optimally assess pericardial disease. Cardiac and pericardial morphology are evaluated by dark-blood T1-weighted fast spin-echo and bright-blood cine steady-state free-precession (SSFP) imaging. Cine SSFP imaging has become the reference sequence to assess and quantify cardiac volumes, myocardial mass, and ventricular function. When acquired in real-time, this sequence can be used to assess ventricular coupling by assessing the changes in ventricular septal shape and motion over the respiratory cycle. Tissue characterization of the heart and pericardium is achieved by dark-blood T1-weighted and dark-blood T2-weighted, short-tau inversion-recovery (STIR) spin-echo imaging, cine SSFP imaging and T1-weighted contrast-enhanced and/or late contrast-enhanced (LCE) imaging following intravenous administration of paramagnetic gadolinium chelates. The LGE sequence uses an inversion recovery pre-pulse to increase image contrast and is well suited to visualize pericardial inflammation. Ventricular inflow and venous flow patterns can be evaluated using phase contrast imaging. < … > The normal pericardium appears on T1-weighted imaging as a thin hypointense (‘dark’) curvilinear structure surrounded by hyperintense (‘bright’) mediastinal and epicardial fat. Normal pericardial thickness ranges from 1.2 to 1.7 mm. < … > It should be emphasized that CMR can accurately distinguish between mixed myopericardial diseases such as mixed inflammatory forms (e.g. myopericarditis or perimyocarditis) and post-myocardial infarction pericardial injury. In patients with constrictive pericarditis, CMR is particularly important in the diagnosis of atypical presentations, such as those with minimally thickened pericardium or effusive-constrictive pericarditis, and those with potentially reversible or transient forms of constrictive pericarditis, showing enhancement of the pericardial layers at LGE imaging. Compared with CT, CMR has the advantage of providing information with regard to the hemodynamic consequences of the non-compliant pericardium on cardiac filling and has the potential of showing fibrotic fusion of pericardial layers. In patients with congenital pericardial pathology and pericardial malignancy, CMR shares the advantages of CT, but allows better tissue characterization and the possibility of evaluating the functional consequences. Moreover, novel techniques, such as diffusion weighted and dynamic contrast-enhanced magnetic resonance imaging, open perspectives for improved tissue characterization in patients with pericardial tumours.”

“A modern approach for the management of pericardial diseases should include the integration of different imaging modalities in order to improve the diagnostic accuracy and clinical management of patients.” (Multimodality imaging). Table 12 of the original guideline summarizes the diagnostic contribution of the different imaging modalities in various pericardial diseases and Table 13 of the guideline compares the value of the different non-invasive imaging modalities to study the pericardium, with CMR being mostly rated as good (++) or excellent (+++).

In addition to this general statement, CMR is repeatedly mentioned in the chapters about specific scenarios. Evidence of pericardial inflammation by CMR is one of the diagnostic criteria for inflammatory pericardial syndrome. In recurrent pericarditis, “CMR may provide confirmatory findings to support the diagnosis in atypical or doubtful cases showing pericardial inflammation through evidence of oedema and contrast enhancement of the pericardium.” In pericarditis associated with myocardial involvement (myopericarditis), CMR can be used to assess impairment of left ventricular function and is recommended “for the confirmation of myocardial involvement and to rule out ischemic myocardial necrosis in the absence of significant coronary disease.” In pericardial effusion, “CMR provide < s > a larger field of view, allowing the detection of loculated pericardial effusion and pericardial thickening and masses, as well as associated chest abnormalities.” In constrictive pericarditis, “imaging evidence of pericardial inflammation by contrast enhancement on CT and/or CMR may be helpful to identify patients with potentially reversible forms of constriction where empiric anti-inflammatory therapy should be considered and may prevent the need for pericardiectomy.” “The utility of CMR in constrictive pericardial disease is well established, providing the opportunity not only to evaluate pericardial thickness, cardiac morphology, and function, but also for imaging intrathoracic cavity structures, allowing the differentiation of constrictive pericarditis from restrictive cardiomyopathy. Assessment of ventricular coupling with real-time cine magnetic resonance during free breathing allows an accurate evaluation of ventricular interdependence and septal bounce.” In post-cardiac injury syndromes, “CMR can be used to show the presence of concomitant pericardial inflammation.” In pericardial involvement in neoplastic disease, “CMR may reveal mediastinal widening, hilar masses and pleural effusion.” The workup of pericardial cysts eventually includes “CMR to define the size, density and neighbouring structures.”

##### Comparison between the current and the last guideline

There is no previous guideline version.

#### 2015 ESC Guidelines for the management of infective endocarditis [26]

##### CMR in specific recommendations

There is no specific recommendation for CMR in infective endocarditis.

##### CMR in the guideline text

Within the subchapter about myocarditis and pericarditis as complications of infective endocarditis, CMR is mentioned to assess myocardial involvement.

##### Comparison between the current and the last guideline

The referral to CMR in the context of suspected myocarditis is new in the 2015 guideline compared to the version from 2009.

#### 2014 ESC Guidelines on diagnosis and management of hypertrophic cardiomyopathy [27]

##### CMR in specific recommendations

This guideline contains 7 recommendations with referral to CMR (Table 20): 1 × I B, 1 × 1 C, 1 × IIa B, 2 × IIa C, 2 × IIb C.

**Table 20:** Recommendations for CMR in the guidelines on diagnosis and management of hypertrophic cardiomyopathy

Recommendation	Class	Level
*Recommendations for cardiovascular magnetic resonance evaluation in hypertrophic cardiomyopathy (HCM)*		
It is recommended that CMR studies be performed and interpreted by teams experienced in cardiac imaging and in the evaluation of heart muscle disease	I	C
In the absence of contraindications, CMR with LGE is recommended in patients with suspected HCM who have inadequate echocardiographic windows, in order to confirm the diagnosis	I	B
In the absence of contraindications, CMR with LGE should be considered in patients fulfilling diagnostic criteria for HCM, to assess cardiac anatomy, ventricular function, and the presence and extent of myocardial fibrosis	IIa	B
CMR with LGE imaging should be considered in patients with suspected apical hypertrophy or aneurysm	IIa	C
CMR with LGE imaging should be considered in patients with suspected cardiac amyloidosis	IIa	C
CMR with LGE may be considered before septal alcohol ablation or myectomy, to assess the extent and distribution of hypertrophy and myocardial fibrosis	IIb	C
*Recommendations on routine follow-up*		
CMR may be considered every 5 years in clinically stable patients, or every 2–3 years in patients with progressive disease	IIb	C

*CMR*  cardiovascular magnetic resonance, *LGE*  Late Gadolinium Enhancement

##### CMR in the guideline text

“CMR embraces several modalities that provide detailed information on cardiac morphology, ventricular function and myocardial tissue characteristics. CMR evaluation of patients with known or suspected HCM should be <...> performed and interpreted by teams experienced in cardiac imaging and in the evaluation of heart muscle disease.”

“CMR should be considered in patients with HCM at their baseline assessment if local resources and expertise permit. In patients with good echocardiographic images, CMR provides similar information on ventricular function and morphology, but it is helpful in establishing the diagnosis of HCM in patients with poor acoustic windows or when some LV regions are poorly visualized—such as the anterolateral wall, the LV apex and the right ventricle. As in 2D echocardiography, over-estimation of wall thickness can result from oblique sections (particularly at the LV apex) or from inclusion of paraseptal structures such as the moderator band or false tendons. Over-estimation of wall thickness is also possible in spoiled gradient echo images and so steady-state free precession (SSFP) cine sequences are preferred. CMR is superior to transthoracic echocardiography in the measurement of LV mass, but LV mass itself correlates weakly with maximal wall thickness and can be normal in patients with asymmetric HCM, especially when it involves less than two LV segments. CMR is superior to standard 2D echocardiography in the detection of LV apical and anterolateral hypertrophy, aneurysms and thrombi, and is more sensitive in the detection of subtle markers of disease, such as myocardial crypts and papillary muscle abnormalities in patients with sarcomeric protein gene mutations. Phase velocity flow mapping sequences can be used to determine the peak velocity of blood flow through the LV outflow tract in patients with LV outflow tract obstruction (LVOTO), but proper alignment of the imaging plane, to obtain the highest flow velocities is time-consuming and prone to error. Intravoxel dephasing and signal loss due to phase offset errors, also make the accurate quantification of turbulent flow difficult and LV outflow gradients can only be measured at rest. <...> In selected cases where echocardiographic images are suboptimal, CMR is helpful in pre-operative planning for surgical myectomy, particularly in patients with multi-level LV obstruction (LV outflow tract and mid-cavity) and in patients with right ventricular (RV) outflow tract abnormalities. CMR can also quantify the amount of tissue necrosis induced by septal alcohol ablation, as well as the location of scarring and the regression of LV mass following the procedure.”

“By using the intrinsic magnetic properties of different tissues and the distribution of gadolinium-based contrast agents, CMR can be used to detect expansion of the myocardial interstitium caused by fibrosis. Late gadolinium enhancement (LGE) is present in 65% of patients (range 33–84%), typically in a patchy mid-wall pattern in areas of hypertrophy and at the anterior and posterior RV insertion points. LGE is unusual in nonhypertrophied segments except in advanced stages of disease, when full-thickness LGE in association with wall thinning is common. LGE may be associated with increased myocardial stiffness and adverse LV remodeling and the extent of LGE is associated with a higher incidence of regional wall motion abnormalities. LGE varies substantially with the quantification method used and the 2-standard deviation technique is the only one validated against necropsy. Assessment of LGE before invasive treatment of LVOTO may be useful in selecting the most appropriate therapy by assessing the degree of septal fibrosis.” “Septal ablation may be less effective in patients with extensive septal scarring on CMR.”

“The association between LGE and long-term outcomes has been examined in several studies <...>. The pooled data support a relationship between LGE and cardiovascular mortality, heart failure death and all-cause death, but show only a trend towards an increased risk of sudden cardiac death (SCD). LGE is associated with NSVT on Holter monitoring. On balance, the extent of LGE on CMR has some utility in predicting cardiovascular mortality, but current data do not support the use of LGE in prediction of SCD risk.”

“CMR rarely distinguishes the causes of HCM by their magnetic properties alone, but the distribution and severity of interstitial expansion can, in context, suggest specific diagnoses”, for example Anderson-Fabry disease, cardiac amyloidosis and athletes heart.

##### Comparison between the current and the last guideline

There is no previous guideline version.

#### 2014 ESC Guidelines on the diagnosis and treatment of aortic diseases [28]

##### CMR in specific recommendations

This guideline contains 9 recommendations with referral to CMR: 8 × I C, 1 × IIa C (Table 21).

**Table 21:** Recommendations for CMR in the guidelines on the diagnosis and treatment of aortic diseases

Recommendation	Class	Level
*Recommendations on diagnostic work-up of acute aortic syndrome*		
In stable patients with a suspicion of acute aortic syndrome (AAS), the following imaging modalities are recommended (or should be considered) according to local availability and expertise: CT (1C), MRI (1C), TOE (IIa C)	I	C
In case of initially negative imaging with persistence of suspicion of AAS, repetitive imaging (CT or MRI) is recommended	I	C
In case of uncomplicated Type B aortic dissection (AD) treated medically, repeated imaging (CT or MRI) during the first days is recommended	I	C
*Recommendations on the management of intramural haematoma*		
In uncomplicated Type B intramural hematoma (IMH), repetitive imaging (MRI or CT) is indicated	I	C
*Recommendations on management of penetrating aortic ulcer*		
In uncomplicated Type B penetrating aortic ulcer (PAU), repetitive imaging (MRI or CT) is indicated	I	C
*Recommendations for the management of aortic root dilation in patients with bicuspid aortic valve*		
Cardiac MRI or CT is indicated in patients with bicuspid aortic valve (BAV) when the morphology of the aortic root and the ascending aorta cannot be accurately assessed by TTE	I	C
In the case of aortic diameter > 50 mm or an increase > 3 mm/year measured by echocardiography, confirmation of the measurement is indicated, using another imaging modality (CT or MRI)	I	C
*Recommendations for follow-up and management of chronic aortic diseases*		
Contrast CT or MRI is recommended, to confirm the diagnosis of chronic aortic dissection	I	C
For follow-up after (T)EVAR in young patients, MRI should be preferred to CT for magnetic resonance compatible stent grafts, to reduce radiation exposure	IIa	C

*CMR*  cardiovascular magnetic resonance, *CT*  computed tomography, *MRI*  magnetic resonance imaging, *TOE*  transesophageal echocardiography, *TTE*  transthoracic echocardiography, *(T)EVAR*  (thoracic) endovascular aortic repair

##### CMR in the guideline text

“With its ability to delineate the intrinsic contrast between blood flow and vessel wall, MRI is well suited for diagnosing aortic diseases. The salient features necessary for clinical decision-making, such as maximal aortic diameter, shape and extent of the aorta, involvement of aortic branches in aneurysmal dilation or dissection, relationship to adjacent structures, and presence of mural thrombus, are reliably depicted by MRI.” “MRI does not require ionizing radiation or iodinated contrast and is therefore highly suitable for serial follow-up studies in (younger) patients with known aortic disease. MRI of the aorta usually begins with spin-echo black blood sequences to outline its shape and diameter and depicting an intimal flap in the presence of aortic dissection (AD). Gradient-echo sequences follow in stable patients, demonstrating changes in aortic diameters during the cardiac cycle and blood flow turbulences—for instance, at entry/re-entry sites in AD, distal to bicuspid valves, or in aortic regurgitation. Contrast-enhanced MRI with intravenous gadolinium can be performed rapidly, depicting the aorta and the arch vessels as a 3D angiogram, without the need for ECG-gating. Gadolinium-enhanced sequences can be performed to differentiate slow flow from thrombus in the false lumen (FL). < … > Time-resolved 3D flow-sensitive MRI, with full coverage of the thoracic aorta, provides the unique opportunity to visualize and measure blood flow patterns. Quantitative parameters, such as pulse wave velocities and estimates of wall shear stress can be determined.” On a scale from “ + ” to “ +  +  + ”, the ease of use of CMR is graded as “ +  + ”, diagnostic reliability as “ +  +  + ”, serial examinations as “ +  +  + ”, and aortic wall visualization as “ +  +  + “.

In acute aortic dissection (AAD), “CT, CMR and TOE are equally reliable for confirming or excluding the diagnosis of AAD. However, CT and MRI have to be considered superior to TOE for the assessment of AAD extension and branch involvement, as well as for the diagnosis of intramural haematoma (IMH), penetrating aortic ulcer (PAU), and traumatic aortic lesions.” “MRI is considered the leading technique for diagnosis of AD, with a reported sensitivity and specificity of 98%. It clearly demonstrates the extent of the disease and depicts the distal ascending aorta and the aortic arch in more detail than is achieved by TOE. The localization of entry and re-entry is nearly as accurate as with TOE and the sensitivity for both near to 90%. The identification of the intimal flap by MRI remains the key finding, usually seen first on spin-echo black-blood sequences. The true lumen (TL) shows signal void, whereas the false lumen (FL) shows higher signal intensity indicative of turbulent flow. MRI is also very useful for detecting the presence of pericardial effusion, aortic regurgitation, or carotid artery dissection. The proximal coronary arteries and their involvement in the dissecting process can be clearly delineated. Flow in the FL and TL can be quantified using phase contrast cine-MRI or by tagging techniques.”

In uncomplicated Type B aortic dissection, “repetitive imaging is necessary, preferably with MRI or CT.” In intramural hematoma, “MRI can be a valuable problem-solving tool, especially when dynamic cine gradient-echo sequences are applied. MRI may also provide a determination of the age of a hematoma, based on the signal characteristics of different degradation products of hemoglobin.” For “long-term surveillance in traumatic aortic injury, MRI is the best alternative for surveillance when magnetic resonance-compatible stent grafts are employed.” In suspected thoracic aortic aneurysms, “based on echocardiography and/or chest X-ray, CT or MRI (with or without contrast) is required to adequately visualize the entire aorta and identify the affected parts.” “When rates of progression have an impact on the therapeutic decision, they should be assessed using alternative techniques (e.g. TTE and CT or MRI) and their consistency checked”. In abdominal aortic aneurysm (AAA), “because of technical improvements, their relatively non-invasive nature and lower cost, CT and MRI have emerged as the current ‘gold standards’ in the pre-operative and post-operative evaluation of AAAs.” In genetic diseases affecting the aorta, “the management of adult women with Turner syndrome associates imaging (echocardiogram and thoracic MRI) with cardiovascular risk assessment.” In Loeys-Dietz syndrome, “a vertebral tortuosity index-measured on a volume-rendered angiogram obtained by thoracic contrast-enhanced MRI—was proposed”. In “aortic diseases associated with bicuspid aortic valve (BAV)”, “in every newly diagnosed patient with BAV, the aortic root and ascending aorta should be visualized with TTE alone or associated with another imaging modality, preferably MRI. < … > In cases of an increase in diameter > 3 mm/year or a diameter > 45 mm measured on TTE, a measurement with another imaging modality (MRI or CT) is indicated. From a diameter of 45 mm, annual follow-up of the ascending aorta is advised. If TTE cannot reliably visualize the ascending aorta, annual imaging with MRI (or CT if MRI is not possible) is indicated.” In coarctation of the aorta, “MRI and CT are the preferred noninvasive techniques to evaluate the entire aorta in adults. Both depict site, extent, and degree of the aortic narrowing, the aortic arch, the pre- and post-stenotic aorta, and collaterals. Both methods detect complications such as aneurysms, re-stenosis, or residual stenosis.” In thromboembolic aortic disease, “MRI can give details on the composition of plaques.” In atherosclerotic aortic occlusion “imaging techniques (CT or MRI) yield more detailed information that can guide the planning of treatment.” In suspected giant cell aortitis, “echocardiography, CT, or MRI are recommended. A thickened aortic wall on CT or MRI indicates inflammation of the aortic wall, and thus active disease.” In Takayasu arteritis, “Echocardiography, MRI, and CT are useful in demonstrating homogeneous circumferential thickening of the aortic wall with a uniform smooth internal surface. < … > Compared with echography, CT and MRI provide better assessment of the entire aorta and its proximal branches, as well as distal pulmonary arteries that are sometimes affected. MRI may show arterial wall edema, a marker of active disease”. In patients with peripheral or splanchnic emboli, “contrast-enhanced MRI of the thoracic and abdominal aorta should be performed, as this investigation is the most sensitive diagnostic tool for detection of an aortic tumor.” In chronic aortic dissection, “diagnosis has to be confirmed by cross-sectional imaging such as contrast-enhanced CT, TOE, or MRI.” “„For imaging follow-up after TEVAR < … > to avoid exposure to radiation, MRI may be more widely used in the future.”

##### Comparison between the current and the last guideline

There is no previous guideline version.

## Discussion

This analysis summarizes the representation of CMR in the 27 currently valid ESC guidelines. It covers 23 new guidelines and 4 guidelines that were already included in the last analysis from 2015 [1]. This update underlines that apart from one single guideline (dyslipidemia), all other 26 guidelines (96.3%) mention CMR as an important diagnostic tool within the topic of the guideline. Nineteen out of the 27 (70.4%) guidelines even include CMR in their tables that provide specific instructions with class of recommendation and level of evidence when to perform CMR. Compared to the last analysis from 2015, the number of explicit recommendations for CMR has increased from 63 to now 92, making up a growth of 146%. Especially the class-II-recommendations have increased from 24 in the 2015 analysis to 47 in the current analysis. The other seven guidelines, though not containing specific recommendations in tables, comprise in part extensive text passages about the value of CMR. The lack of table-recommendations in these guidelines should not be regarded as downgrading the value of CMR compared to the guidelines with specific recommendations. The ESC strives to achieve one common standard in all ESC guidelines. Nevertheless, the decision to put content in tables or in text is not always uniform.

The number of recommendations that refer to cardiomyopathies has clearly increased from 7 in the analysis from 2015 to 21 in the current analysis. In addition, the number of recommendations with regard to myocarditis has increased from 2 to 5, and referring to myocardial tissue characterization in general from 2 to 12. Like the 2015 analysis, most recommendations have evidence level C.

In general, this analysis confirms that CMR is established within the ESC as an integral part of the diagnostic modalities. It demonstrates that this acceptance has grown during over the past years, based on clinical experiences, expert opinions and new evidence created by the scientific community active in the field of CMR.

Thereby, CMR has particularly grown its important role in the field of cardiomyopathies and myocardial tissue characterization for making a diagnosis and for assessing the risk of a disease. The guideline about ventricular arrhythmia and sudden cardiac death published in 2022 numerically contains the most guideline recommendations referring to CMR. In DCM, HCM and AD, CMR is often regarded as a mandatory part of the diagnostic algorithms. During the work-up of suspected myocarditis, CMR is also rated in several guidelines as extremely important and often regarded as mandatory, e.g. in the guideline about ventricular arrhythmia and sports cardiology. Also, the newly established guideline about cardio-oncology puts CMR in the center to early detect myocardial inflammation due to cardiotoxic effects of some tumor therapies. The use of multi-parametric CMR as proposed in the updated Lake-Louise criteria published in 2018 [29] further improves the diagnostic performance of CMR to detect any inflammatory state of the myocardium and/or pericardium. This advance underlines the important role of CMR for identifying different entities of myocardial abnormalities.

In the field of coronary artery disease, which has a high practical and economic impact due to the high disease prevalence, CMR is constantly positioned equivalently to other non-invasive tests regarding ischemia testing. Regarding the differentiation of the disease in suspected MINOCA, CMR newly became the central diagnostic tool to separate ischemic from non-ischemic / inflammatory myocardial diseases. Even though widely used in clinical practice, viability testing with CMR (and any other imaging technique) still plays a minor role in the guidelines due to inconclusive evidence regarding its benefit for patient management.

Even though the total number of CMR recommendations across all guidelines increased underlining the importance of CMR, there are some single guidelines with a decreasing number of specific CMR recommendations (arterial hypertension, myocardial revascularization, chronic coronary syndrome). This observation was rather attributed to the mode of presentation of content than to a downgrading of the role of CMR. Furthermore, there was no explicit downgrading regarding the class of recommendation and level of evidence of a single recommendation between guideline versions.

As a converse conclusion from this analysis, not performing CMR in certain scenarios must increasingly be regarded as not compliant with guideline recommendations. However, in reality, access to CMR is still limited in many regions. The causes are multifactorial, including issues of costs and reimbursement as well as skills and training. The governments and health care providers that decide about reimbursement and access to diagnostics must be aware that they take the responsibility for enabling or preventing guideline-based diagnostic approaches for the individual patient. From the physicians’ perspective, structured training programs must be established, and further studies are needed to continue demonstrating the impact of CMR on patient management. From the industry perspective, attempts to reduce the complexity and costs of CMR in general, to accelerate and simplify the acquisition of CMR images and to automate image interpretation including artificial intelligence is needed to reduce costs and thus to improve the access to CMR. Facilitated access to CMR could also contribute to realize important clinical trials that are currently performed slowly or could not performed. New evidence could further support CMR examinations to appear in class I A recommendations.

## Conclusions

CMR is represented in almost every ESC guideline as a valuable diagnostic tool. The guideline tables contain many specific recommendations for the use of CMR in certain scenarios. Compared to the last analysis from 2015, the extent of recommendations for CMR has grown. To enable the physicians to manage their patients in accordance with the ESC guidelines, access to CMR must be realized.

## Supplementary Information

**Additional file 1. Table S1.** CMR in adults with congenital heart disease.

## Data Availability

The datasets used and/or analyzed during the current study are available from the corresponding author on reasonable request.

## Abbreviations

AAA: Abdominal aortic aneurysm
AAD: Acute aortic dissection
AD: Arrhythmogenic disease
ALVC: Arrhythmogenic left ventricular cardiomyopathy
ARVC: Arrhythmogenic right ventricular cardiomyopathy
BAV: Bicuspid aortic valve
CABG: Coronary artery bypass grafting
CAD: Coronary artery disease
CCTA: Cardiac computed tomography angiography
CMR: Cardiovascular magnetic resonance
DCM: Dilated cardiomyopathy
DM: Diabetes mellitus
EF: Ejection fraction
EMB: Endomyocardial biopsy
COPD: Chronic obstructive pulmonary disease
CPET: Cardiopulmonary exercise testing
CT: Computed tomography
ECG: Electrocardiography
ESC: European Society for Cardiology
FL: False Lumen
HCM: Hypertrophic cardiomyopathy
HNDCM: Hypokinetic non-dilated cardiomyopathy
ICD: Implantable cardioverter defibrillator
IMH: Intramural hematoma
LAD: Left anterior descending coronary artery
LGE: Late Gadolinium enhancement
LV: Left ventricle
LVNC: Left ventricular non compaction
MRA: Magnetic resonance angiography
MRI: Magnetic resonance imaging
PAU: Penetrating aortic ulcer
PET: Positron emission tomography
PCI: Percutaneous coronary intervention
RV: Right ventricle
SCD: Sudden cardiac death
SPECT: Single photon emission computed tomography
STEMI: ST elevation myocardial infarct
TEVAR: Thoracic endovascular aortic repair
TL: True lumen
TOE: Transoesophageal echocardiography
TTE: Transthoracic echocardiography
VA: Ventricular arrhythmia
VT: Ventricular tachycardia
