# Supplementary material for: Cardiovascular magnetic resonance in the guidelines of the European Society of Cardiology: a comprehensive summary and update
Source: J Cardiovasc Magn Reson. 2023 Jul 24;25:42. doi: 10.1186/s12968-023-00950-z (PMC10364363; doi:10.1186/s12968-023-00950-z)
Supplement: Supplementary file 1 — Additional file 1. Table S1. CMR in adults with congenital heart disease. [file 12968_2023_950_MOESM1_ESM.docx]

**Additional material**

Table S1. CMR in adults with congenital heart disease

| **Specific lesion** | **Role of CMR** |
| --- | --- |
| Atrial septal defect and anomalous pulmonary venous connection | Sinus venosus defects in general require TOE for accurate diagnosis. CMR/CCT is an alternative and superior in case of inferior sinus venosus defects). CMR is rarely required but may be useful for assessment of RV volume overload, identification of inferior sinus venosus defect, quantification of pulmonary to systemic flow ratio (Qp:Qs), and evaluation of pulmonary venous connection. |
| Ventricular septal defect | CMR can serve as an alternative if echocardiography is insufficient, particularly for assessment of LV volume overload and  shunt quantification. |
| Atrioventricular septal defect | CMR is indicated when additional quantification of ventricular  volumes and function, AV valve regurgitation, or intracardiac  shunting is required for decision making. |
| Patent ductus arteriosus | CMR is indicated when additional quantification of LV volumes  and quantification of shunt (Qp:Qs) is needed. CMR/CCT can further evaluate the anatomy where required. |
| Valvular aortic stenosis | CMR/CCT, despite having potential for assessing aortic stenosis, is mainly required to assess dilation of the ascending aorta, in cases where measurement is unreliable with echocardiography.  CMR or CCT of the aorta is recommended in patients with a native BAV, patients with a history of isolated valve replacement where the ascending aorta is not well visualized on TTE, and in patients with root/ascending diameters >40mm. |
| Supravalvular aortic stenosis | CMR/CCT is useful for detailed evaluation of supravalvular anatomy, in particular, when multilevel LVOTO is present or for  (pre-operative) assessment of coronary artery anatomy and other aortic or aortic branch lesions (e.g. carotid and renal artery  stenosis), and central and branch PAs.  Lifelong and regular follow-up, including echocardiography, is  required to determine progression of obstruction (rare), LV size/  function, and development of symptoms, as well as after surgery to detect late restenosis, development of aneurysm (CMR/CCT), and the occurrence or progression of CAD. |
| Subaortic stenosis | CMR may be useful to characterize complex LVOTO anatomies,  especially in patients with poor acoustic window. |
| Coarctation of the aorta | CMR and CCT, including 3D reconstruction, are the preferred  non-invasive techniques to evaluate the entire aorta in adolescents and adults. Both depict site, extent, and degree of the aortic narrowing, the aortic arch and head and neck vessels, the pre and post-stenotic aorta, and collaterals. Both methods detect  complications such as aneurysms, false aneurysms, restenosis, or residual stenosis. All CoA patients require regular follow-up at least every year. Imaging of the aorta (preferably with CMR) is required to document post-repair or post-interventional anatomy and complications (restenosis, aneurysm, false aneurysm formation). |
| Marfan syndrome and related heritable thoracic  aortic diseases | CMR or CCT angiography from head to pelvis should be performed in every patient at baseline, providing imaging of the  entire aorta and branching vessels. Lifelong and regular multidisciplinary follow-up at an expert center is required. Echocardiography and CCT/CMR are the principal examinations. |
| Right ventricular outflow tract  obstruction | For quantification of RV sizes, volumes, and EF, CMR is a more robust and more reliable technique <than echocardiography>. CMR and CCT frequently provide additional important information identifying the level(s) of obstruction, including at the subinfundibular (DCRV), conduit, or branch PA levels, and assessment of RV volumes, pulmonary annulus, outflow tract and artery dimensions, and differential pulmonary blood flow. CMR and CCT are the methods of choice for visualization of pulmonary dilation and peripheral PS. |
| Ebstein anomaly | CMR has value with regards to prognostication, and for evaluation before and after surgery, as it offers unrestricted views for assessment and quantification of the dilated right heart, RV function, and TV function. |
| Tetralogy of Fallot | CMR is the method of choice for assessment of RV volume and  function; PR; size, shape, and expansion of the PAs; infundibulum; the ascending aorta; the position of great vessels or conduits in relation to the sternum (resternotomy); and evaluation for residual shunt (Qp:Qs). Late gadolinium enhancement demonstrates fibrosis, the extent of which relates to other risk factors for VT and SCD. T1mapping may have an emerging role.  Follow-up evaluation needs to look for the complications. All patients should have CMR at regular intervals, dependent on the pathology found. |
| Pulmonary atresia with ventricular septal defect | CMR, CCT, and cardiac catheterization are required to determine sources of pulmonary blood supply and size of PAs, to assess MAPCAs, and obtain haemodynamics. In repaired patients, CMR is used for requirements similar to patients with TOF [for RV volumes and function, PR, size, shape, and expansion of the PAs, and the size of the ascending aorta, and for residual shunt (Qp:Qs)]. |
| Transposition of the great arteries | CMR provides more reliable and more robust quantitative assessment of systemic RV systolic function than echocardiography, and of patency of the atrial baffles. Size of the great arteries can be measured reliably; an abnormally wide PA and/or large subpulmonary LV may indicate PH. Shunt related to baffle leak can be quantified (Qp:Qs). Late gadolinium enhancement in the systemic RV predicts clinical outcome. |
| Arterial switch operation | CMR provides more reliable quantitative assessment of ventricular volumes, EF, and neo-aortic dilatation or regurgitation. Pulmonary trunk and branches can be visualized, together with their relation to the (dilated) neo-aortic root. Flow distribution between left and right lung can be calculated. Stress CMR is an alternative technique to assess myocardial perfusion and possible |
| Rastelli-type operation | CMR provides a more robust quantification of LV and RV volumes, aortic diameters, and EF. The RV-PA conduit, often difficult to visualize by echocardiogram, and peripheral PAs can be readily seen and measured with CMR. In the presence of a residual VSD, Qp:Qs can be calculated. |
| Congenitally corrected transposition of the great arteries | CMR provides intracardiac and great vessel anatomy and is indicated for quantification of ventricular volumes, mass, and EF, especially since echocardiographic assessment of systolic function in systemic RVs is difficult and less reliable. |
| Right ventricular to pulmonary  artery conduit | CMR is used to quantify conduit stenosis and/or regurgitation, RV volumes and mass, and to assess PAs. CMR/CCT is helpful for coronary artery anatomy and proximity of the RV/conduit, and other structures to the retro sternum. |
| Univentricular heart | CMR is the imaging modality of choice for extracardiac anatomy,  including veno-atrial and ventriculo-arterial connections (CCT is  an alternative). Detailed morphological information of intracardiac  anatomy can also be obtained. CMR is also the method of  choice for quantification of ventricular volumes, EF, and relative  distribution of blood flow in the left and right lungs. CMR and an exercise test are required at least once at adult age and at further intervals timed according to baseline findings. |
| Patients after Fontan operation | CMR is helpful for evaluation of the Fontan pathway, collaterals,  and pulmonary veins (e.g. right pulmonary vein obstruction by  enlarged RA) and for thrombus. <…> CMR is regularly performed  for ventricular volumes, Fontan pathway patency and flows, to  evaluate AV valve regurgitation, subaortic obstruction, myocardial  fibrosis, and for detection of thrombus.  Cardiac catheterization <… and> Integration with CMR for flows  (cardiac output) may allow more precise measurement of PVR. |
| Coronary anomalies | Non-pharmacological functional imaging (e.g. nuclear study, echocardiography, or CMR with physical stress) is recommended in patients with coronary anomalies to confirm/ exclude myocardial ischemia. |

CMR = Cardiovascular magnetic resonance, TOE = transesophageal echocardiography, CCT = cardiac computed tomography, RV = right ventricular, LV = left ventricular, AV = atrioventricular, BAV = bicuspid aortic valve, TTE = transthoracic echocardiogaphy, LVOTO = left ventricular outflow tract obstruction, PA = pulmonary artery, CAD = coronary artery disease, 3D = three dimensional, CoA = coarctation of the aorta, EF = ejection fraction, DCRV = Double-chambered right ventricle, PS = pulmonary stenosis, TV = tricuspid valve, PR = pulmonary regurgitation, VT = ventricular tachycardia, SCD = sudden cardiac death, MAPCAs = major aortic pulmonary collaterals, TOF = tetralogy of Fallot, PH = pulmonary hypertension, VSD = ventricular septal defect, PVR = pulmonary vascular resistance
